# Oxygenated Acyclic Diterpenes with Anticancer Activity from the Irish Brown Seaweed *Bifurcaria bifurcata*

**DOI:** 10.3390/md18110581

**Published:** 2020-11-23

**Authors:** Vangelis Smyrniotopoulos, Christian Merten, Daria Firsova, Howard Fearnhead, Deniz Tasdemir

**Affiliations:** 1 School of Chemistry, National University of Ireland Galway, University Road, H91 TK33 Galway, Ireland; vsmy@hotmail.com (V.S.); dashafirsova63@gmail.com (D.F.); 2 Organische Chemie 2, Ruhr-Universität Bochum, Universitätsstraße 150, 44801 Bochum, Germany; christian.merten@ruhr-uni-bochum.de; 3 Pharmacology and Therapeutics, School of Medicine, National University of Ireland Galway, University Road, H91 W2TY Galway, Ireland; howard.fearnhead@nuigalway.ie; 4 GEOMAR Centre for Marine Biotechnology (GEOMAR-Biotech), Research Unit Marine Natural Product Chemistry, GEOMAR Helmholtz Centre for Ocean Research Kiel, Am Kiel-Kanal 44, 24106 Kiel, Germany; 5 Faculty of Mathematics and Natural Sciences, Kiel University, Christian-Albrechts-Platz 4, 24118 Kiel, Germany

**Keywords:** *Bifurcaria bifurcata*, linear diterpene, brown alga, seaweed, VCD, anticancer activity

## Abstract

Brown alga *Bifurcaria bifurcata* is a prolific source of bioactive acyclic (linear) diterpenes with high structural diversity. In the continuation of our investigations on Irish brown algae, we undertook an in-depth chemical study on the *n*-hexanes and chloroform subextracts of *B. bifurcata* that led to isolation of six new (**1**–**6**) and two known (**7**–**8**) acyclic diterpenes. Chemical structures of the compounds were elucidated by a combination of 1D and 2D NMR, HRMS, FT-IR, [α]_D_ and vibrational circular dichroism (VCD) spectroscopy. Compounds **1**–**8**, as well as three additional linear diterpenes (**9**–**11**), which we isolated from the same seaweed before, were tested against the human breast cancer cell line (MDA-MB-231). Several compounds moderately inhibited the growth of the MDA-MB-231 cell line with IC_50_ values ranging from 11.6 to 32.0 μg/mL. The present study carried out on the lipophilic extracts of the Irish *B. bifurcata* shows the enormous capacity of this seaweed to produce a large palette of acyclic diterpenes with diverse oxygenation and substitution patterns and promising bioactivities.

## 1. Introduction

*Bifurcaria bifurcata* R. Ross is a brown alga belonging to the family *Sargassaceae* (order *Fucales*). Due to its morphology (cylindrical thallus, rhizoidal holdfast and regular narrow forking), it is regarded as a cylindrical brown seaweed and named as brown forking weed or brown tuning fork weed [1,2]. It is found abundantly in the rocky shores of Northern Atlantic coast, stretching from Morocco to France, Southwest Britain and Western Ireland [1,3]. Chemically, *B. bifurcata* is a prolific seaweed, representing over 8% of the total metabolites of the family *Sargassaceae* [3,4]. Although fatty acids, steroids, carotenoids, phlorotannins, phenolic acids and flavonoids have been reported from this species [5,6,7], the acyclic diterpenes obtained from the lipophilic extracts of the seaweed represent the largest natural product class. These compounds originate from geranylgeraniol and bear a C16 backbone with four double bonds and five olefinic methyl groups. A wide array of substitutions, e.g., oxygenation at C-1, C-6, C-7, C-12 or C-13, or other rearrangements, such as different unsaturation patterns, terminal furan, butenolide or hydroxybutenolide ring formation, middle chain hydroxylations or isomerisation, lead to a wide diversity of acyclic diterpenes with high structural and stereochemical diversity. These intriguing compounds serve as chemotaxonomical markers and exhibit a wide array of ecological activities [2,3,8,9]. A number of pharmacological activities, e.g., antimicrobial, antiprotozoal, antitubercular, anti-inflammatory, anticancer and neuroprotective, have been reported from *B. bifurcata* extracts and acyclic diterpenes isolated therefrom [2,6,10,11,12].

We previously reported antiprotozoal and antimycobacterial activities of the Irish *B. bifurcata* [13] as well as the isolation and absolute configuration (AC) determination of the known acyclic diterpenes eleganediol (9) and bifurcane (10) and the new compound bifurcatriol (11) [14,15]. In the continuation of our interest in this brown algal species, we continued our chemical analyses on the *n*-hexanes and chloroform subextracts of *B. bifurcata* that led to the isolation of eight linear diterpenoids. Compounds **1**–**6** are new acyclic diterpenes, while **7**–**8** were previously reported from *B. bifurcata* [9,16]. Herein, we report the isolation and structure elucidation of **1**–**8** and the anticancer activity of Compounds **1**–**11**.

## 2. Results and Discussion

The freeze-dried fronds of *B. bifurcata* were successively extracted with CH_2_Cl_2_ and MeOH. A solvent–solvent extraction of the combined organic extract by a modified Kupchan partition scheme afforded the *n*-hexanes, CHCl_3_ and aqueous subextracts. The *n*-hexanes and CHCl_3_ subextracts were subjected to automated flash chromatography and RP-HPLC to yield compounds **1**–**8**. 

Compound **1** was isolated as a colorless oil. The molecular formula C_20_H_32_O_3_ was assigned based on a sodium adduct *m/z* 343.2247 [M + Na]^+^ observed in its HRESIMS spectrum, indicating five double bond equivalents (DBEs). Its IR spectrum contained absorption bands typical for hydroxyl, conjugated carboxylic acid and alkene functions (*ν*_max_ 3369, 1690 and 1642 cm^−1^, respectively). The ^1^H NMR spectrum of **1** (Table 1) revealed the presence of four methine protons at δ_H_ 5.06 (t, *J* = 5.5 Hz, H-6), δ_H_ 5.13 (br. d, *J* = 8.4 Hz, H-14), δ_H_ 5.18 (t, *J* = 6.7 Hz, H-10) and δ_H_ 5.66 (br. s, H-2) and five olefinic methyl singlets at δ_H_ 1.70 (H_3_-16), δ_H_ 1.67 (H_3_-17), δ_H_ 1.63 (H_3_-18), δ_H_ 1.58 (H_3_-19) and δ_H_ 2.15 (H_3_-20), plus an oxymethine proton at δ_H_ 4.38 (td, *J* = 8.4, 5.3 Hz, H-13). The ^13^C NMR spectrum (Table 2) contained 20 carbon resonances, which, in conjunction with the *g*HSQC spectrum, were sorted out into a carbonyl group (δ_C_ 169.1, C-1), four quaternary sp^2^, four sp^2^ methine, one sp^3^ oxymethine, five sp^3^ methylene and four tertiary methyl carbons. The comparison of these data with those of the known compound eleganediol (**9**) [14] indicated that **1** is a linear diterpene containing an OH substitution, four double bonds and a carboxylic acid group, fulfilling the required number (5) of DBEs within **1**.

In order to confirm and the position of the carboxylic acid and the OH substitution, and thereby the planar structure of **1**, we ran *g*COSY and *g*HMBC experiments. Three short spin systems were visible in the COSY spectrum. The first two proton networks included two methylene and one olefinic methine protons each, i.e., H_2_-4/H_2_-5/H-6 (fragment a) and H_2_-8/H_2_-9/H-10 (fragment b). The third network consisted of H_2_-12, the oxymethine proton (H-13, δ_H_ 4.38) and the olefinic methine H-14, yielding the fragment c. The obtained COSY fragments a–c were readily connected through ^1^H-^13^C HMBC correlations between H-6/C-8 and H_3_-19/C-6; H-10/C-12 and H-10/CH_3_-18, and from terminal methyl groups H_3_-16 and H_3_-17 to both C-14 and C-15. The significantly downfield resonances of C-3 (δ_C_ 162.6), H_3_-20 (δ_H_ 2.15) and H-2 (δ_H_ 5.66), as well as the appearance of H-2 as an isolated broad singlet, suggested the presence of a terminal carboxylic acid at C-1. Further evidence for the location of the carboxylic acid at C-1 was provided by the diagnostic HMBC cross peaks between H-2/C-1, H_3_-20/C-1 and H_3_-20/C-2, plus a W coupling of H-2 with H_2_-4 in the COSY spectrum. This finalised the gross structure of compound **1**. 

The NOESY spectrum of **1** assisted in the identification of the *E* geometry of all double bonds, on the basis of key NOE couplings observed between H-2/H_2_-4 (for C-2), H-6/H_2_-4, H-6/H_2_-8 (for C-6), H-10/H_2_-8, H-10/H_2_-12 (for C-10) and finally between H-14/H_2_-12 (for C-14). The assignment of H_3_-16 as *pro*-*E* and H_3_-17 as *pro*-*Z* was possible due to additional NOE correlations between H-14/H_3_-16 and H-13/H_3_-17, respectively. The absolute configuration of the only stereogenic center at C-13 was assigned by vibrational circular dichroism (VCD) spectroscopy. We have shown in a previous VCD study on eleganediol (**9**) and bifurcane (**10**) [14] that the C-13 stereocenter gives rise to a distinct VCD spectroscopic signature which is independent of the substitution at C-1. The experimental VCD spectrum of **1** showed the same spectral pattern as observed for **9** (Supplementary Materials Figure S42), which led us to conclude without further theoretical spectra analysis that the AC of **1** is also 13*S.*

The molecular formula C_20_H_34_O_3_ was assigned to compound **2** by its HRESIMS data (*m/z* 345.2398 [M + Na]^+^). Compound **2** was identified as possessing a 13*S*-hydroxygeranylgeraniol scaffold with high similarities to eleganediol (**9**). However, 1D NMR and the *g*HSQC spectra indicated that one of the vinylic methyl signals (CH_3_-17) was replaced with a primary alcohol group (δ_H_ 3.99 d and δ_H_ 4.25 d, *J* = 12.4 Hz; δ_c_ 62.3, t). These methylene protons only showed geminal coupling with each other on the *g*COSY spectrum. The HMBC spectrum that contained correlations between H_3_-16/C-17; H_2_-17/C-14 and H_2_-17/C-16 clearly proved that the H_3_-17 methyl group was converted to a hydroxymethylene function, completing the structure of **2**. The all-*E* geometry of the double bond carbons and *S* configuration of C-13 were confirmed by the characteristic ^13^C and ^1^H resonances of **2** that were similar to those of compounds **1** and **9** [14,15]. 

**Figure 1:**
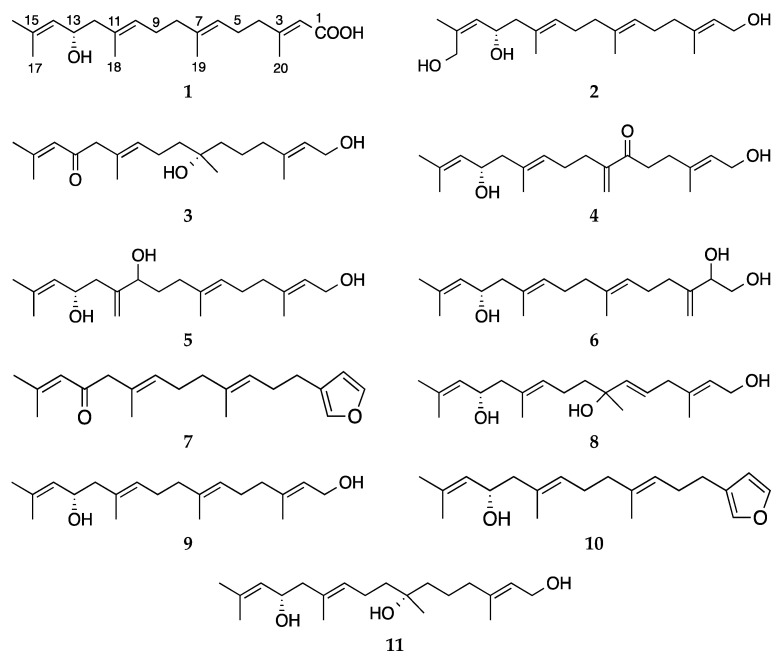
Chemical structures of linear diterpenes **1**–**11**.

The HRESIMS analysis of compound **3** revealed a molecular formula C_20_H_34_O_3 (_*m/z* 345.2402 [M + Na]^+^). The detailed inspection of the 1D and 2D NMR data of compound **3** indicated that it was an analogue of bifurcatriol (**11**), which is 13*S*,7*S*-dihydroxygeranylgeraniol [15]. The only difference between the two compounds was the conversion of the C-13-OH functionality in **11** to a ketone group (δ_C_ 197.7) in **3**, which was also confirmed by the IR absorption band at *ν*_max_ 1703 cm^−1^. The oxidation at C-13 led to deshielding of H-14 (δ_H_ 5.96), H_3_-17 (δ_H_ 2.12) and H_2_-12 (δ_H_ 2.97, 2H), with the latter resonating as an isolated singlet (Table 1). The position of the C-13 ketone group was verified by the key HMBC correlations observed between H_2_-12/C-13 and H-14/C-13. The inspection of its full 2D NMR data (DEPT-HSQC, COSY, HMBC and NOESY) supported the suggested structure of the ketoalcohol **3**, as shown in Figure 1. An *S* configuration was assigned to C-7 based on comparison of the 1D NMR data of **3** with those of bifurcatriol (**11**) [15], the C-13-hydroxy derivative of **3**.

Compound **4** was isolated as a colorless oil. The HRESIMS of **4** returned a sodium adduct ion at *m/z* 343.2246 [M + Na]^+^ in agreement with a molecular formula C_20_H_32_O_3_ requiring five DBEs. A careful investigation of its 1D and 2D NMR spectra indicated that **4** lacked one of the olefinic methyl signals, but contained instead two broad singlets at δ_H_ 5.99 and δ_H_ 5.72, and a CH_2_ signal at δ_C_ 124.2 (t). Compound **4** had identical NMR data to those of **1** and **9** regarding the three double bonds (∆^2,3^, ∆^10,11^ and ∆^14,15^) and four olefinic methyl groups (CH_3_-16, CH_3_-17, CH_3_-18 and CH_3_-20), hence CH_3_-19 must have converted into an exomethylene group, to fulfil the fourth DBE. On the other hand, the notably deshielded resonance of H_3_-19 and H_2_-5 (δ_H_ 2.79 dd, *J* = 7.9, 7.5 Hz, Table 1) suggested the presence of a strong electronegative group in their vicinity. The ^13^C signal at δ_C_ 201.2 (C-6) and the FT-IR absorption band at *ν*_max_ 1718 cm^−1^ of **4** supported the presence of a ketone group either at C-6 or C-8. The latter option (C-8-oxo) was ruled out, as H_2_-8 was part of a proton network composed of H-10 (δ_H_ 5.19), H_2_-9 (δ_H_ 2.14) and H_2_-8 (δ_H_ 2.30) in the *g*COSY spectrum of **4** (Figure S26). Thus, the ketone group was assigned to C-6. An in-depth analysis of the cross peaks in the HMBC spectrum of **4**, particularly those between H_2_-8/C-19, H-9/C-8, H_2_-19/C-7 and H_2_-19/C-8 lent proof for the presence of the exomethylene function at C-19, while further HMBC correlations from H_2_-19, H_2_-4, H_2_-5 and H_2_-8 to C-6 corroborated the position of the oxo function at C-6. Identical NMR data around the only stereocenter C-13 was indicative of the same *S* configuration at C-13.

Compound **5** had a molecular formula C_20_H_34_O_3_ deduced by its HRMS data (*m/z* 345.2405 [M + Na]^+^). Analysis of the NMR spectra indicated that **5** is another 13*S*-hydroxygeranylgeraniol derivative comprising four olefinic methyl groups. It was evident from two singlets at δ_H_ 4.95 and δ_H_ 5.08 (1H each) that correlated with the CH_2_ signal at δ_C_ 114.3 on the *g*HSQC spectrum that one of the methyl groups (CH_3_-18) was converted into an exomethylene group in **5**. Furthermore, NMR signals at δ_H_ 4.06 (dd, *J* = 7.8, 5.5 Hz, H-10) and δ_c_ 74.9 (C-10) suggested the presence of an additional secondary alcohol group (Table 1 and Table 2). The HMBC spectrum contained key correlations from H-10 and H_2_-12 to C-18; from H_2_-18 to C-10, C-11 and C-12; and finally from H_2_-8 and H_2_-9 to C-10 assigned, respectively, the exocyclic double bond to C-18 and the second OH group to H-10. Due to rapid decomposition of the compound **5** during the NOESY experiment, the stereochemistry at C-10 could not be assigned. The all-*E* geometry of the double bond carbons and *S* configuration of C-13 were confirmed by their characteristic ^13^C and ^1^H NMR resonances [14,15].

As deduced by HREIMS (*m/z* 345.2396 [M + Na]^+^), the new compound **6** was determined as a positional isomer of compound **5** with the same molecular formula C_20_H_34_O_3_. The only difference between the two compounds was the position of both the exocyclic double bond (δ_H_ 4.95 s, δ_H_ 5.13 s; δ_c_ 111.0 t) and the additional secondary OH group (δ_H_ 4.16 dd, *J* = 7.2, 3.4 Hz, δ_c_ 74.9). Thanks to the HMBC correlations observed between (i) from H_2_-20 to C-2, C- 3 and C-4, (ii) from H-2 to C-20, and from H_2_-4 to C-20 and (iii) from H-2 to C-1 and C-3, the olefinic methylene group was assigned to C-20 and the secondary alcohol to C-2. Similar to **5**, compound **6** had low stability and decomposed during the final 2D NMR experiment (NOESY), hence the stereochemistry of C-2 remains unassigned.

In vitro anticancer activity of the isolated compounds was evaluated against the breast cancer cell line MDA-MB-231. The only compounds that displayed activity were the new compound **1** (IC_50_ 30.7 μg/mL) and the known compounds **9** (IC_50_ 11.6 μg/mL) and **10** (IC_50_ 32.0 μg/mL). Compounds **7, 8** and **11** were inactive even at the highest test concentrations (100 μg/mL), while the remaining new compounds **2**–**6** were not tested due to availability of minute amounts or stability issues.

The present study has shown the Irish brown alga *B. bifurcata* to contain a wide array of linear diterpenes. Oxygenation in multiple positions is common in many acyclic diterpenes obtained from this seaweed [2,3,16,17,18]. Notably, all compounds isolated herein possessed oxygenation at C-13 accompanied by additional hydroxy, ketone, carboxylic acid substitutions or a terminal furan ring. We have previously applied VCD spectroscopy for identification of the absolute configuration (*S*) of linear diterpenes from this seaweed, e.g., eleganediol (**9**) and bifurcane (**10**) [14], with a single stereocenter (C-13) as well as for bifurcatriol (**11**) that bears two stereocenters (C-7, C-13) [15]. In the present study, it was again successfully applied to confirm the (S)-configuration of C-13 in compound **1**. By structural analogy, NMR data comparison and biosynthetic considerations, we easily assigned 13*S* stereochemistry to the other new compounds **2**–**6**.

Numerous algal metabolites belonging to different natural product classes have been shown to exert anticancer activity [19,20]. The halogenated monoterpene halomon or the highly oxygenated triterpene laurenmariannol represent well-known examples of seaweed-derived terpenes with low to submicromolar level cytotoxicity [19], indicating the understudied potential of seaweeds. As for *B. bifurcata*, several C-12 hydroxy-bearing linear diterpenes have been reported with such potential and their potency [19,20,21,22] is generally similar to C-13 OH-bearing acyclic diterpenes isolated in this study.

In summary, the current study implies the diversity of polyoxygenated acyclic diterpenes in Irish brown alga *B. bifurcata*. Only three compounds showed modest anticancer activity against human breast cancer cells (MDA-MB-231). Several compounds could not be assessed for their bioactivity due to stability issues or low amounts available. Some compounds obtained in large amounts previously (e.g., **9** and **10**) [14] can be submitted to semi-synthesis studies for enhancement of their anticancer potency and assessment of their further biological and ecological activities.

## 3. Materials and Methods

### 3.1. General Experimental Procedures

Optical rotations were determined with a Unipol L1000 Schmidt + Haensch polarimeter at the sodium D line (589.3 nm), at 20 °C, with a 10 cm cell. UV spectra were acquired in spectroscopic grade CHCl_3_ or MeOH on a Varian, Cary 100 UV-Vis spectrophotometer. IR spectra were recorded on a Perkin Elmer 400 or a Perkin Elmer Spectrum One ATR FT-IR spectrometer. NMR spectra were acquired using a Jeol 400 MHz, a Varian 500 MHz or an Agilent 600 MHz spectrometer. Chemical shifts are given on the δ (ppm) scale, referenced to the residual solvent signal (CDCl_3_: δ_H_ 7.24, δ_C_ 77.0 or C_6_D_6_: δ_H_ 7.16, δ_C_ 128.0), and *J* values in Hz. High-resolution mass spectrometric data were measured on an Agilent QTOF 6540 MS system, with electrospray ionisation (ESI) in the positive ion mode, coupled to an Agilent 1290 Infinity UPLC system, operating on the elution gradient: 50% B for 8 min, increasing to 100% B in 3 min, maintaining 100% B for 5 min (solvent A: H_2_O + 0.1% formic acid, solvent B: MeCN + 0.1% formic acid), on a Zorbax Eclipse Plus C18 RRHD (50 × 2.1 mm, 1.8 μm) column, at 0.5 mL/min, with UV detection at 200−600 nm. Thin layer chromatography (TLC) was performed with Kieselgel 60 F254 aluminium support plates (Merck) and spots were detected after spraying with 6% vanillin and 15% H_2_SO_4_ in MeOH reagent. All solvents were of HPLC or LCMS grade and were purchased from Sigma-Aldrich.

### 3.2. Algal Material

*Bifurcaria bifurcata* was collected from the intertidal rock pool at Kilkee, Co. Clare of Ireland, in May 2009. A voucher specimen is kept at the Herbarium of the Biodiscovery Laboratory in the Irish Marine Institute (BDV0015).

### 3.3. Extraction and Isolation

The algal thalli were cleaned with sea water. CH_2_Cl_2_ and MeOH were used for exhaustive extraction of the freeze-dried material (132.4 g dry weight) at room temperature. The organic extracts were combined and evaporated to dryness on a rotary evaporator. The resulting dark green paste (12.0 g) was subjected to a modified Kupchan liquid–liquid partition. Briefly, the crude extract was dissolved in 90% MeOH (200 mL) and partitioned against *n*-hexanes (3 × 200 mL). The water concentration was increased to 35%, before partitioning against CHCl_3_. Evaporation of the solvents under reduced pressure and temperature afforded *n*-hexanes (3.6 g) and CHCl_3_ subextracts (7.6 g).

An aliquot of the *n*-hexanes subextract (2.3 g) was chromatographed by flash column chromatography over silica gel using a 10% gradient of EtOAc in hexane to afford nine combined fractions (**H1**–**H9**). **Fr. H2** (547 mg) which, eluted with 20% EtOAc, was subjected to fractionation on an Agilent 971FP system loaded with a pre-packed silica column SF15-12g (Agilent), operating at the following gradient: 0% B for 10 min, increasing to 6% B in 50 min, increasing to 10% B in 20 min, increasing to 100% B in 10 min, maintaining 100% B for 15 min (solvent A: *n*-hexanes, solvent B: EtOAc), at a flow of 15 mL/min, to afford **7** (1.2 mg).

The CHCl_3_ subextract (6.8 g) was subjected to gradient flash CC fractionation (Agilent 971FP system, pre-packed Agilent silica column SF25-80g, operating with the following gradient: 0% B for 5 min, to 5% B in 15 min, at 5% B for 10 min, to 10% B in 10 min, at 10% B for 10 min, to 40% B in 40 min, to 100% B in 10 min, at 100% B for 10 min, solvent A: *n*-hexanes, solvent B: EtOAc, flow of 25 mL/min, affording 21 fractions (**C1**–**C21**). **Fr. C4** (43.9 mg) was subjected to RP-HPLC. The separation was conducted using an Agilent 1260 system equipped with a diode array and an ELSD detector (split flow) on a Kromasil 100 C18 5u (250 × 8 mm, 5 μm) HPLC column. The gradient elution using 55% MeCN for 13 min, increasing to 100% MeCN in 5 min and maintaining 100% MeCN for 20 min (solvent A: H_2_O, solvent B: MeCN), at a flow of 1.5 mL/min, afforded **1** (11.2 mg). **Fr. C10** (118.0 mg) was subjected to RP-HPLC on the same system, using the same solvent gradient to afford pure **4** (1.0 mg, t_R_ 25.9 min). **Fr. C12** (66.0 mg) was separated by RP-HPLC under the same conditions to afford pure **8** (1.1 mg, t_R_ 22.7 min), **3** (1.0 mg, t_R_ 25.9 min), **5** (7.2 mg, t_R_ 26.2 min) and **6** (1.7 mg, t_R_ 26.8 min). Finally, the RP-HPLC purification of **fr. C16** (49.0 mg) under the same conditions yielded **2** (0.9 mg).

### 3.4. VCD Spectroscopy

The IR and VCD spectra of **1** were recorded on a Bruker Vertex 70V spectrometer equipped with a PMA 50 module for polarisation-modulated measurements. Samples were held in a sealed BaF_2_ IR cell with 100 µm path length (Specac). Both IR and VCD spectra were recorded at 4 cm^−1^ spectral resolution by accumulating 32 and ~20,000 scans, respectively. Baseline correction of the spectra was done by subtraction of the solvent spectra measured under identical conditions.

Compound **1**: colorless oil; [α]_D_ +9.4 (*c* 0.32, MeOH), +4.6 (c 1.13, CHCl_3_); UV (CHCl_3_) *λ*_max_ (log *ε*) 243 (2.19) nm; IR (thin film) *ν*_max_ 3369, 2917, 1690, 1642, 1437, 1247, 1157, 1022, 868 cm^−1^; ^1^H NMR (500 MHz, CDCl_3_) and ^13^C NMR (125 MHz, CDCl_3_) see Table 1 and Table 2; HRESIMS *m/z* 343.2247 [M + Na]^+^ (calcd for C_20_H_32_O_3_Na, 342.2244).

Compound **2**: colorless oil; [α]_D_ −12.7 (*c* 0.34, CHCl_3_); UV (CHCl_3_) *λ*_max_ (log *ε*) 233 (2.02) nm; IR (thin film) *ν*_max_ 3422, 2936, 2872, 1656, 1459, 1385, 1193, 1060 cm^−1^; ^1^H NMR (500 MHz, CDCl_3_) and ^13^C NMR (125 MHz, CDCl_3_) see Table 1 and Table 2; HRESIMS *m/z* 345.2398 [M + Na]^+^ (calcd for C_20_H_34_O_3_Na, 345.2400).

Compound **3**: colorless oil; [α]_D_ +6.0 (*c* 0.10, MeOH); UV (CHCl_3_) *λ*_max_ (log *ε*) 245 (2.23) nm; IR (thin film) *ν*_max_ 3468, 2932, 1703, 1022 cm^−1^; ^1^H NMR (600 MHz, C_6_D_6_) and ^13^C NMR (150 MHz, C_6_D_6_) see Table 1 and Table 2; HRESIMS *m/z* 345.2402 [M + Na]^+^ (calcd for C_20_H_34_O_3_Na, 345.2400).

Compound **4**: colorless oil; [α]_D_ −1.6 (*c* 1.11, CHCl_3_); UV (CHCl_3_) *λ*_max_ (log *ε*) 246 (2.26) nm; IR (thin film) *ν*_max_ 3492, 2957, 2874, 1718, 1456, 1369, 1240, 1030, 897, 753 cm^−1^; ^1^H NMR (500 MHz, CDCl_3_) and ^13^C NMR (125 MHz, CDCl_3_) see Table 1 and Table 2; HRESIMS *m/z* 343.2246 [M + Na]^+^ (calcd for C_20_H_32_O_3_Na, 343.2244).

Compound **5**: colorless oil; [α]_D_ −12.7 (*c* 0.34, CHCl_3_); UV (CHCl_3_) *λ*_max_ (log *ε*) 236 (2.18) nm; IR (thin film) *ν*_max_ 3377, 2942, 2861, 1378, 1022 cm^−1^; ^1^H NMR (500 MHz, CDCl_3_) and ^13^C NMR (125 MHz, CDCl_3_) see Table 1 and Table 2; HRESIMS *m/z* 345.2405 [M + Na]^+^ (calcd for C_20_H_34_O_3_Na, 345.2400).

Compound **6**: colorless oil; [α]_D_ −3.1 (*c* 1.09, CHCl_3_); UV (CHCl_3_) *λ*_max_ (log *ε*) 234 (1.98) nm; IR (thin film) *ν*_max_ 3448, 3225, 2952, 1683, 1454, 1378, 1019 cm^−1^; ^1^H NMR (600 MHz, CDCl_3_) and ^13^C NMR (150 MHz, CDCl_3_) see Table 1 and Table 2; HRESIMS *m/z* 345.2396 (calcd for C_20_H_34_O_3_Na, 345.2400).

### 3.5. Anticancer Activity Assessments

The breast cancer cell line MDA-MB-231 (ATCC) was maintained in Dulbecco’s modified Eagle’s medium (DMEM, Sigma-Aldrich) supplemented with 10% fetal bovine serum and 1% penicillin/streptomycin (Sigma-Aldrich) and incubated at 37 °C, 5% CO_2_. MDA-MB-231 cells were seeded in a 96-well plate (1 × 10^4^ cells per well) and cultured for 24 h (37 °C, 5% CO_2_) before being treated with extracts at a final concentration of 0–100 μg/mL (vehicle control: 1% DMSO; positive control: 10 μM 5-Fluorouracil). After 72 h, cell viability was assessed using Alamar Blue assay. Briefly, 40 μL Alamar Blue (0.56 mM) was added to each well containing 200 μL of cell culture medium (93 μM final Alamar Blue concentration). After 6 h incubation, the fluorescence of each well was assessed (λ_ex_ = 530 nm; λ_em_ = 595 nm) using a Victor 3V 1420 multilabel counter. Cell viability was calculated and expressed as a percentage of untreated control cells. The data are the mean ± SD of three experiments and GraphPad Prism software was used to plot the data and to determine the IC_50_ values.

## Figures and Tables

**Table 1:** ^1^H NMR data of **1**–**6** (CDCl_3_, 500 MHz), δ_H_ values in ppm, *J* in Hz.

C	1	2	3 ^a,b^	4	5	6 ^a^
1	-	4.12, br. d (6.8)	3.99, d (6.7)	4.12, br. d (6.8)	4.11, d (6.9)	3.66, dd (11.2, 3.4) 3.53, dd (11.2, 7.2)
2	5.66, br. s	5.40 br. t (6.8)	5.40, br. t (6.7)	5.38, br. t (6.8)	5.37, br. t (6.9)	4.16, dd (7.2, 3.4)
3	-	-	-	-	-	-
4	2.18, m	2.03, m	1.90, br. t (7.5)	2.31, m	2.05, m	2.10, m 2.04, m
5	2.17, m	2.10, m	1.41, m	2.79, dd (7.9, 7.5)	2.12, m	2.18, m
6	5.06, t (5.5)	5.07, br. t (6.6)	1.30, m	-	5.13, br. t (6.9)	5.09, br. t (6.7)
7	-	-	-	-	-	-
8	2.00, t (7.1)	2.02 m	1.42, m	2.30 m	2.03, m	2.05, m
9	2.13, m 2.10, m	2.16 m	2.08, m	2.14 m	1.62, m	2.12, m
10	5.18, t (6.7)	5.20, br. t (6.9)	5.27, br. t (7.1)	5.19, br. t (6.7)	4.06, dd (7.8, 5.5)	5.17, br. t (6.9)
11	-	-	-	-	-	-
12	2.11, m	2.14 m	2.97, s	2.11, m	2.28, dd (14.3, 9.5) 2.16, dd (14.3, 3.3)	2.11, m
13	4.38, td (8.4, 5.3)	4.44 td (7.8, 4.5)	-	4.39, td (8.4, 7.2)	4.42, ddd (9.5, 8.5, 3.3)	4.36, ddd (9.3, 8.3, 4.0)
14	5.13, br. d (8.4)	5.29 br. d (7.8)	5.96, hept (1.1)	5.13, br. d (8.4)	5.21, dhept (8.5, 1.2)	5.12, dhept (8.3, 1.2)
15	-	-	-	-	-	-
16	1.70, br. s	1.81 br. s	1.47, d (1.1)	1.70, br. s	1.71, d (1.2)	1.70, d (1.2)
17	1.67, br. s	4.25, d (12.4) 3.99, d (12.4)	2.12, d (1.1)	1.67, br. s	1.68, d (1.2)	1.67, d (1.2)
18	1.63, br. s	1.63, br. s	1.69, br. s	1.61, br. s	5.08, s 4.95, s	1.63, br. s
19	1.58, br. s	1.58, br. s	1.02, s	5.99, br. s 5.72, br. s	1.59, br. s	1.59, br. s
20	2.15, br. s	1.65, br. s	1.48, br. s	1.68, br. s	1.64, br. s	5.13, s 4.95, s

Recorded ^a^ at 600 MHz, ^b^ in C_6_D_6_.

**Table 2:** ^13^C NMR data of compounds **1**–**6** (CDCl_3_, 125 MHz).

C	1	2	3 ^a,b^	4 ^c^	5	6 ^a^
1	169.1 s	59.3 t	59.4 t	59.2 t	59.4 t	65.6 t
2	114.5 d	123.5 d	125.0 d	123.7 d	123.5 d	74.9 d
3	162.6 s	139.4 s	138.2 s	138.3 s	139.6 s	148.2 s
4	41.0 t	39.2 t	40.3 t	33.6 t	39.5 t	32.3 t
5	25.8 t	26.0 t	22.3 t	35.8 t	26.2 t	26.0 t
6	123.2 d	124.4 d	41.9 t	201.2 s	123.9 d	124.1 d
7	136.0 s	134.8 s	71.9 s	148.2 s	135.1 s	135.3 s
8	39.4 t	39.4 t	41.8 t	30.9 t	35.5 t	39.4 t
9	26.3 t	26.1 t	23.3 t	26.8 t	33.9 t	26.1 t
10	128.4 d	129.1 d	129.7 d	127.6 d	74.9 d	128.6 d
11	131.7 s	131.4 s	130.1 s	132.4 s	148.7 s	131.7 s
12	48.2 t	48.3 t	55.5 t	48.0 t	40.2 t	48.2 t
13	65.7 d	64.6 d	197.7 s	65.7 d	69.0 d	65.4 d
14	127.3 d	129.6 d	123.3 d	127.4 d	125.2 d	127.0 d
15	135.0 s	139.4 s	154.7 s	135.0 s	136.8 s	135.0 s
16	25.8 q	21.9 q	27.3 q	25.6 q	25.8 q	25.8 q
17	18.2 q	62.3 t	20.6 q	18.0 q	18.2 q	18.2 q
18	16.2 q	16.1 q	16.6 q	16.1 q	114.3 t	16.1 q
19	15.9 q	15.8 q	27.0 q	124.2 t	16.1 q	15.9 q
20	19.1 q	16.3 q	16.1 q	16.3 q	16.2 q	111.0 t

Recorded ^a^ at 150 MHz, ^b^ in C_6_D_6_. ^c^ Chemical shifts (^13^C) were extracted from *g*HSQC and *g*HMBC spectra.

## Supplementary Materials

The following are available online at https://www.mdpi.com/1660-3397/18/11/581/s1, Supplementary Figures S1–S41: 1D and 2D NMR, HRESIMS and FT-IR spectra of compounds **1**–**6**. Supplementary Figure S42: VCD spectra of **1** and **9**.

## References

[1] *Bifurcaria bifurcata* R.Ross. http://www.seaweed.ie/descriptions/Bifurcaria_bifurcata.php.

[2] Pais A.C.S., Saraiva J.A., Rocha S.M., Silvestre A.J.D., Santos S.A.O. (2019). Current research on the bioprospection of linear diterpenes from *Bifurcaria bifurcata*: From extraction methodologies to possible applications. Mar. Drugs.

[3] Muñoz J., Culioli G., Köck M. (2013). Linear diterpenes from the marine brown alga *Bifurcaria bifurcata*: A chemical perspective. Phytochem. Rev..

[4] Rindi F., Soler-Vila A., Guiry M.D., Hayes M. (2012). Taxonomy of marine macroalgae used as sources of bioactive compounds. Marine Bioactive Compounds: Sources, Characterization and Applications.

[5] Koch M., Glombitza K.W., Rösener H.U. (1981). Polyhydroxyphenyl ethers from *Bifurcaria bifurcata*. Phytochemistry.

[6] Santos S.A.O., Trindade S.S., Oliveira C.S.D., Parreira P., Rosa D., Duarte M.F., Ferreira I., Cruz M.T., Rego A.M., Abreu M.H. (2017). Lipophilic fraction of cultivated *Bifurcaria bifurcata* R. Ross: Detailed composition and in vitro prospection of current challenging bioactive properties. Mar. Drugs.

[7] Agregán R., Munekata P.E.S., Franco D., Dominguez R., Carballo J., Lorenzo J.M. (2017). Phenolic compounds from three brown seaweed species using LC-DAD-ESI-MS/MS. Food Res. Int..

[8] Amico V. (1995). Marine brown algae of family Cystoseiraceae: Chemistry and chemotaxonomy. Phytochemistry.

[9] Ortalo-Magné A., Culioli G., Valls R., Pucci B., Piovetti L. (2005). Polar acyclic diterpenoids from *Bifurcaria bifurcata* (Fucales, Phaeophyta). Phytochemistry.

[10] Freile-Peregrin Y., Tasdemir D. (2019). Seaweeds to the rescue of forgotten diseases: A review. Bot. Marina.

[11] Silva J., Alves C., Freitas R., Martins A., Pinteus S., Ribeiro J., Gaspar H., Alfonso A., Pedrosa R. (2019). Antioxidant and neuroprotective potential of the brown seaweed *Bifurcaria bifurcata* in an in vitro Parkinson’s disease model. Mar. Drugs.

[12] Valls R., Banaigs B., Piovetti L., Archavlis A., Artaud J. (1993). Linear diterpene with antimitotic activity from the brown alga *Bifurcaria bifurcata*. Phytochemistry.

[13] Spavieri J., Allmendinger A., Kaiser M., Casey R., Hingley-Wilson S., Lalvani A., Guiry M., Blunden G., Tasdemir D. (2010). Antimycobacterial, antiprotozoal and cytotoxic potential of twenty-one brown algae (Phaeophyceae) from British and Irish waters. Phytotherapy Res..

[14] Merten C., Smyrniotopoulos V., Tasdemir D. (2015). Assignment of absolute configurations of highly flexible linear diterpenes from the brown alga *Bifurcaria bifurcata* by VCD spectroscopy. Chem. Commun..

[15] Smyrniotopoulos V., Merten C., Kaiser M., Tasdemir D. (2017). Bifurcatriol, a new antiprotozoal acyclic diterpene from the brown alga *Bifurcaria bifurcata*. Mar. Drugs.

[16] Hougaard L., Anthoni U., Christophersen C., Nielsen P.H. (1991). Eleganolone derived diterpenes from *Bifurcaria bifurcata*. Phytochemistry.

[17] Valls R., Piovetti L., Banaigs B., Archavlis A., Pellegrini M. (1995). (S)-13-Hyroxygeranylgeraniol-derived furanoditerpenes from *Bifurcaria bifurcata*. Phytochemistry.

[18] Culioli G., Daoudi M., Mesguiche V., Valls R., Piovetti L. (1999). Geranylgeraniol-derived diterpenoids from the brown alga *Bifurcaria bifurcata*. Phytochemistry.

[19] Lefranc F., Koutsaviti A., Ioannou E., Kornienko A., Roussis V., Kiss R., Newman D. (2019). Algae metabolites: From in vitro growth inhibitory effects to promising anticancer activity. Nat. Prod. Rep..

[20] Kiruba N.J.M., Pradeep M.A., Thatheyus A.J. (2018). Discovering promising anti-cancer drug candidates from marine algae. Sci. Int..

[21] Zee O.P., Kim D.K., Choi S.U., Lee C.O., Lee K.R. (1999). A new cytotoxic acyclic diterpene from *Carpesium divaricatum*. Arch. Pharm. Res..

[22] Culioli G., Ortalo-Magne A., Daoudi M., Thomas-Guyon H., Valls R., Piovetti L. (2004). Trihydroxylated linear diterpenes from the brown alga *Bifurcaria bifurcata*. Phytochemistry.

