# Supplementary material for: Oxygenated Acyclic Diterpenes with Anticancer Activity from the Irish Brown Seaweed Bifurcaria bifurcata
Source: Mar Drugs. 2020 Nov 23;18(11):581. doi: 10.3390/md18110581 (PMC7700283; doi:10.3390/md18110581)

Supplementary Information

Oxygenated Acyclic Diterpenes with Anticancer Activity from the Irish Brown Seaweed *Bifurcaria bifurcata*

Vangelis Smyrniotopoulos [1], Christian Merten [2], Daria Firsova [1,], Howard Fearnhead [3], and Deniz Tasdemir [1,4,5*]

[1] School of Chemistry, National University of Ireland Galway, University Road, Galway, Ireland; vsmy@hotmail.com; daria.firsova@abbvie.com
[2] Ruhr-Universität Bochum, Organische Chemie 2, Universitätsstraße 150, 44801 Bochum, Germany; christian.merten@ruhr-uni-bochum.de
[3] Pharmacology and Therapeutics, School of Medicine, National University of Ireland Galway, University Road, Galway, Ireland; howard.fearnhead@nuigalway.ie
[4] GEOMAR Centre for Marine Biotechnology (GEOMAR-Biotech), Research Unit Marine Natural Product Chemistry, GEOMAR Helmholtz Centre for Ocean Research Kiel, Am Kiel-Kanal 44, 24106 Kiel, Germany
[5] Faculty of Mathematics and Natural Sciences, Kiel University, Christian-Albrechts-Platz 4, 24118 Kiel, Germany

* Correspondence: dtasdemir@geomar.de; Tel.: +49-431-600-4430, ORCID ID: 0000-0002-7841-6271

List of Figures

Figure S1. ^{1}H NMR spectrum of compound **1** (500 MHz, $CDCl_3$).

Figure S2. ^{13}C NMR spectrum of compound **1** (125 MHz, $CDCl_3$).

Figure S3. *g*HSQC spectrum of compound **1** (500/125 MHz, $CDCl_3$).

Figure S4. *g*COSY spectrum of compound **1** (500 MHz, $CDCl_3$).

Figure S5. *g*HMBC spectrum of compound **1** (500/125 MHz, $CDCl_3$).

Figure S6. NOESY spectrum of compound **1** (500 MHz, $CDCl_3$).

Figure S7. HR-ESIMS report of compound **1**.

Figure S8. FT-IR spectrum of compound **1**.

Figure S9. ^{1}H NMR spectrum of compound **2** (500 MHz, $CDCl_3$).

Figure S10. ^{13}C NMR spectrum of compound **2** (125 MHz, $CDCl_3$).

Figure S11. *g*HSQC spectrum of compound **2** (500/125 MHz, $CDCl_3$).

Figure S12. *g*COSY spectrum of compound **2** (500 MHz, $CDCl_3$).

Figure S13. *g*HMBC spectrum of compound **2** (500/125 MHz, $CDCl_3$).

Figure S14. HR-ESIMS report of compound **2**.

Figure S15. FT-IR spectrum of compound **2**.

Figure S16. ^{1}H NMR spectrum of compound **3** (600 MHz, C_6D_6).

Figure S17. ^{13}C NMR spectrum of compound **3** (150 MHz, C_6D_6).

Figure S18. *g*HSQC spectrum of compound **3** (600/150 MHz, C_6D_6).

Figure S19. *g*COSY spectrum of compound **3** (600 MHz, C_6D_6).

Figure S20. *g*HMBC spectrum of compound **3** (600/150 MHz, C_6D_6).

Figure S21. NOESY spectrum of compound **3** (600 MHz, C_6D_6).

Figure S22. HR-ESIMS report of compound **3**.

Figure S23. FT-IR spectrum of compound **3**.

Figure S24. ^{1}H NMR spectrum of compound **4** (500 MHz, $CDCl_3$).

Figure S25. *g*HSQC spectrum of compound **4** (500/125 MHz, $CDCl_3$).

Figure S26. *g*COSY spectrum of compound **4** (500 MHz, $CDCl_3$).

Figure S27. *g*HMBC spectrum of compound **4** (500/125 MHz, $CDCl_3$).

Figure S28. HR-ESIMS report of compound **4**.

Figure S29. FT-IR spectrum of compound **4**.

Figure S30. ^{1}H NMR spectrum of compound **5** (500 MHz, $CDCl_3$).

Figure S31. ^{13}C NMR spectrum of compound **5** (125 MHz, $CDCl_3$).

Figure S32. *g*HSQC spectrum of compound **5** (500/125 MHz, $CDCl_3$).

Figure S33. *g*HMBC spectrum of compound **5** (500/125 MHz, $CDCl_3$).

Figure S34. HR-ESIMS report of compound **5**.

Figure S35. FT-IR spectrum of compound **5**.

Figure S36. ^{1}H NMR spectrum of compound **6** (600 MHz, $CDCl_3$).

Figure S37. ^{13}C NMR spectrum of compound **6** (150 MHz, $CDCl_3$).

Figure S38. *g*COSY spectrum of compound **6** (600 MHz, $CDCl_3$).

Figure S39. *g*HMBC spectrum of compound **6** (600/150 MHz, $CDCl_3$).

Figure S40. HR-ESIMS report of compound **6**.

Figure S41. FT-IR spectrum of compound **6**.

Figure S42. Comparison of the IR/VCD spectra of **1** with those of **9** and calculations.

Figure S1. ^{1}H NMR spectrum of compound **1** (500 MHz, $CDCl_3$)

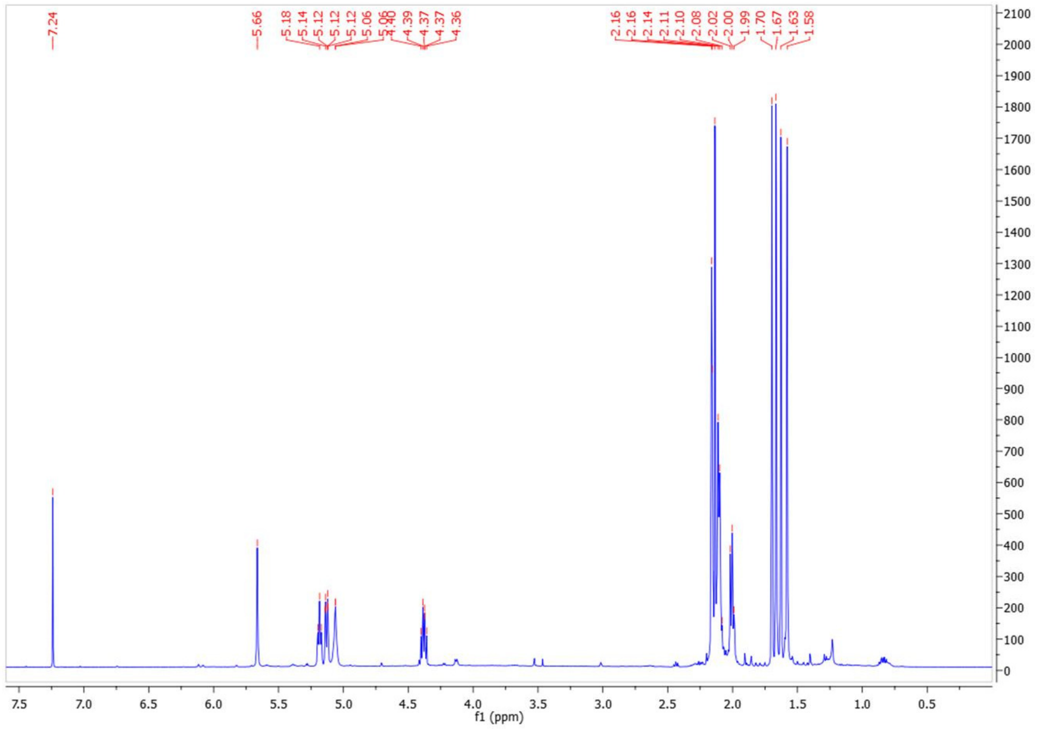

Figure S2. ^{13}C NMR spectrum of compound **1** (125 MHz, $CDCl_3$)

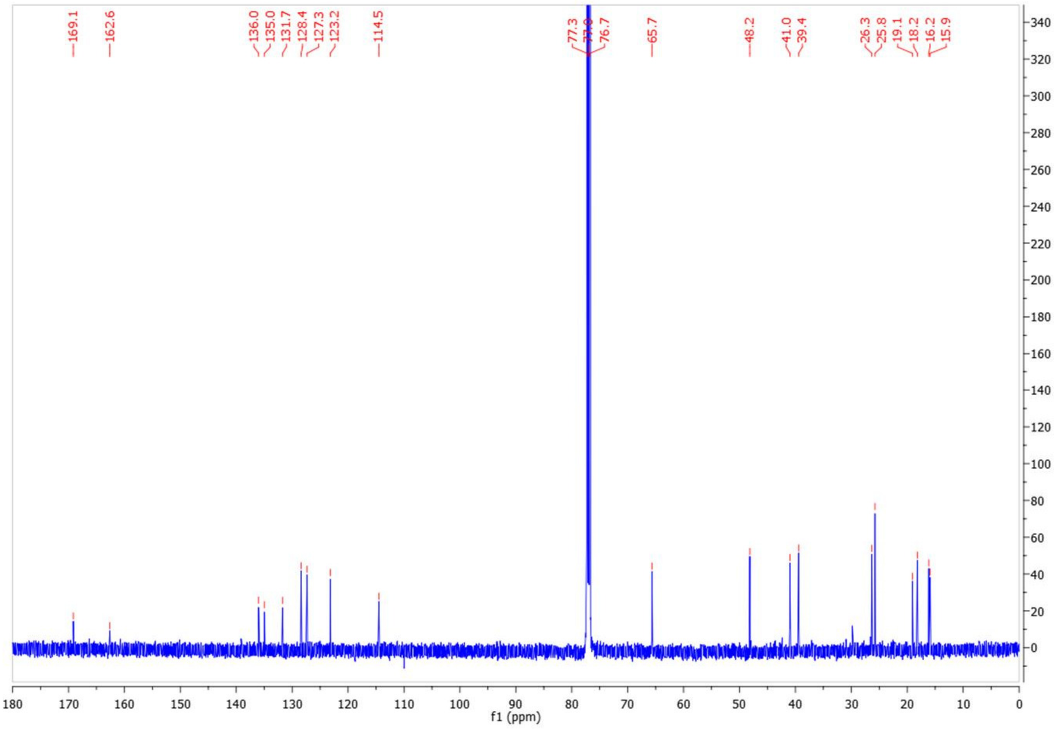

Figure S3. *g*COSY spectrum of compound **1** (500 MHz, $CDCl_3$)

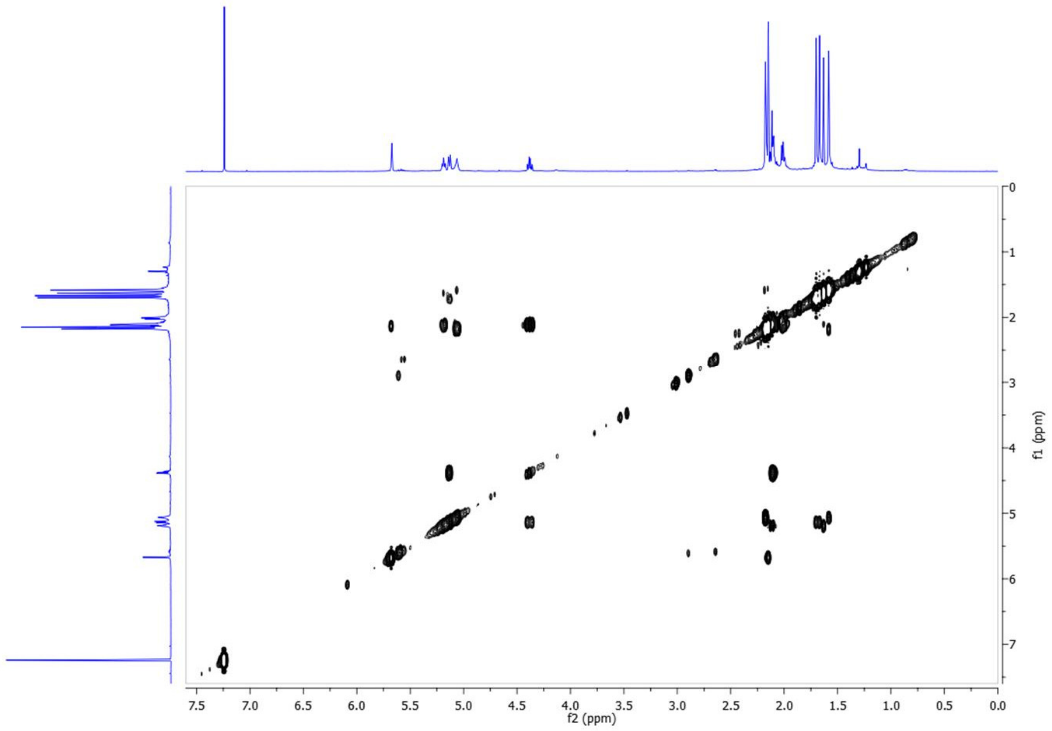

Figure S4. *g*HSQC spectrum of compound **1** (500/125 MHz, $CDCl_3$)

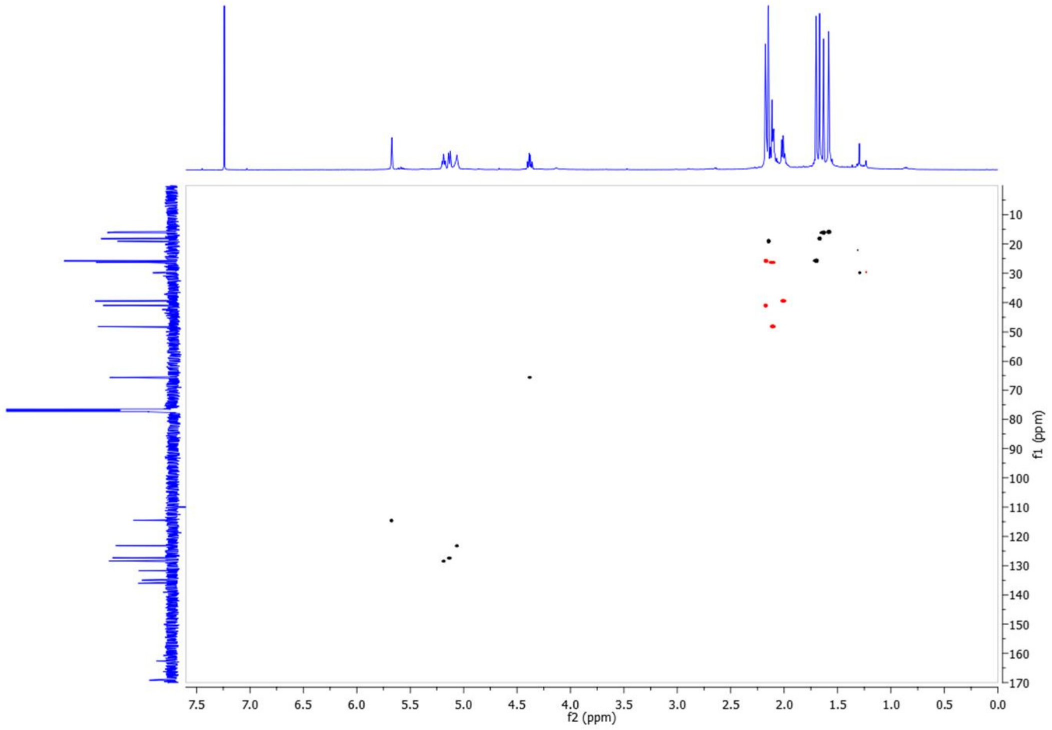

Figure S5. *g*HMBC spectrum of compound **1** (500/125 MHz, CDCl3)

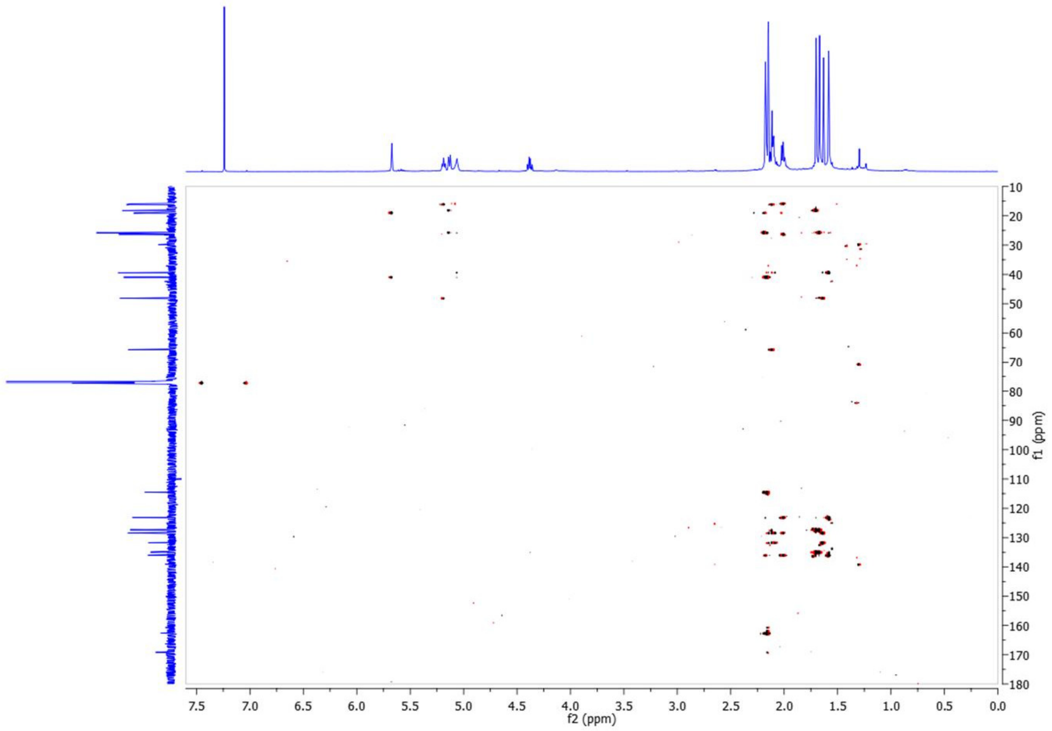

Figure S6. NOESY spectrum of compound **1** (500 MHz, CDCl3)

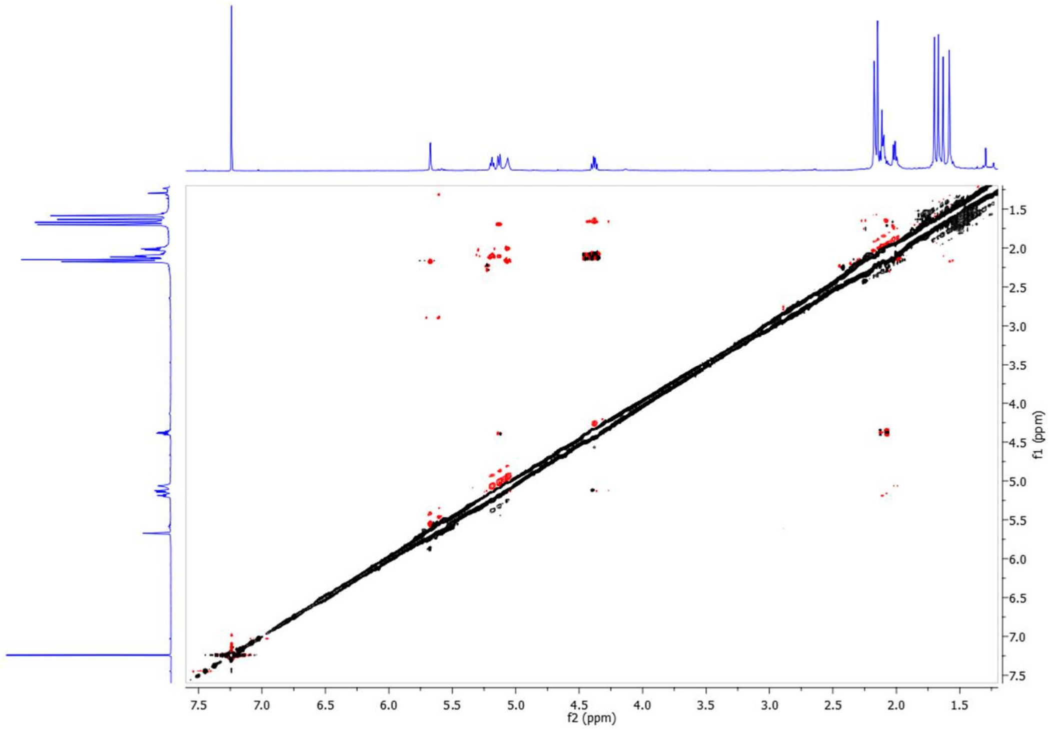

Figure S7. HR-ESIMS report of compound **1**

Qualitative Compound Report

**Data File**	bifu11_15C4-3_2014-07-30_1.d	**Sample Name**	15C4-3_bifu11
**Sample Type**	Sample	**Position**	P1-A4
**Instrument Name**	Instrument 1	**User Name**	
**Acq Method**	VsmyPosFragile_BDV15.m	**Acquired Time**	30-Jul-14 21:44:45
**IRM Calibration Status**	Success	**DA Method**	Default.m
**Comment**			

**Sample Group**	**Info.**
**Fragmentor**	**Nozzle Voltage**

Compound Table

Compound Label	RT	Mass	Abund	Formula	Tgt Mass	Diff (ppm)	MFG Formula	DB Formula
Cpd 1:	7.327	320.2355	458490	C20H32O3	320.2351	1.05	C20H32O3	C20H32O3

Compound Label	*m/z*	RT	Algorithm	Mass
Cpd 1:	987.68	7.327	Find By Formula	320.2355

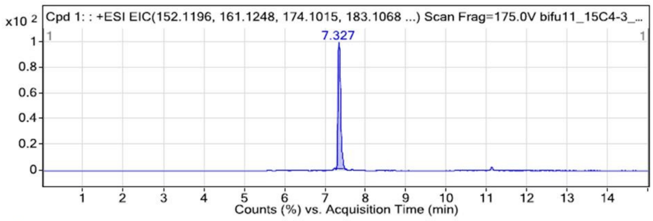

MS Spectrum

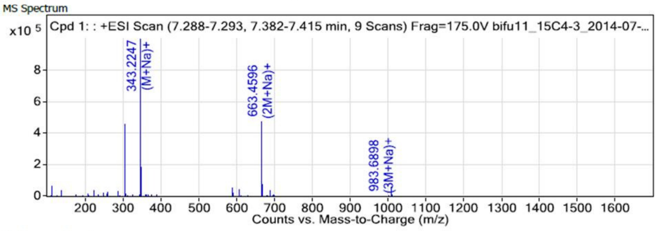

MS Zoomed Spectrum

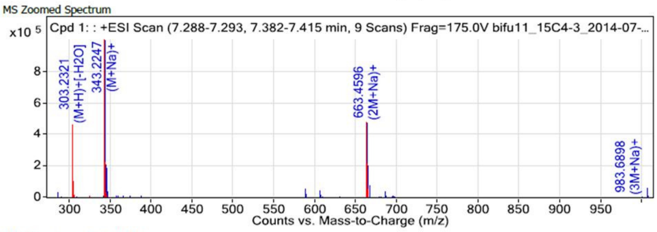

MS Spectrum Peak List

m/z	*Calc m/z*	Diff(ppm)	z	Abund	Formula	Ion
303.2321	303.2319	0.69	1	458490.3	C20H31O2	(M+H)+[-H2O]
321.2421	321.2424	-0.92	1	1865.3	C20H33O3	(M+H)+
325.2132	325.2138	-1.99	1	4429.1	C20H30NaO2	(M+Na)+[-H2O]
343.2247			1	999262.6		
343.2247	343.2244	0.87		999262.6	C20H32NaO3	(M+Na)+
344.2285	344.2278	2.17	1	209145.7	C20H32NaO3	(M+Na)+
345.2417	345.2306	32.16	1	191339.1	C20H32NaO3	(M+Na)+
663.4596	663.4595	0.08	1	478296.8	C40H64NaO6	(2M+Na)+
664.4632	664.4629	0.39	1	204538.3	C40H64NaO6	(2M+Na)+
983.6898	983.6947	-4.95	1	3840.5	C60H96NaO9	(3M+Na)+

--- End Of Report ---

Figure S8. FT-IR spectrum of compound **1**.

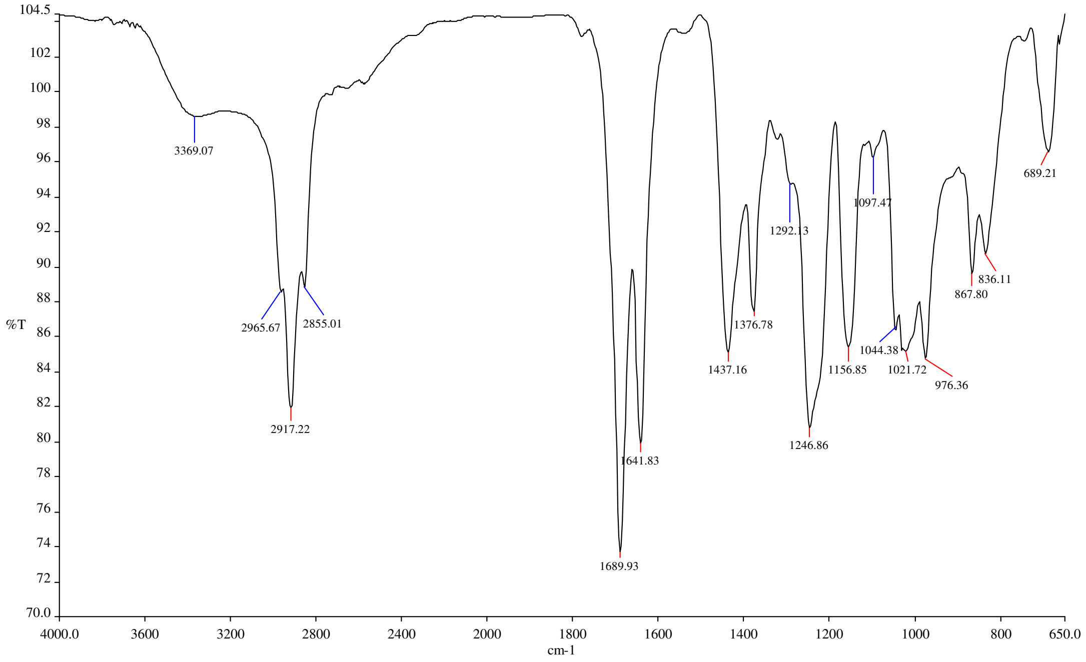

Figure S9. ^{1}H NMR spectrum of compound **2** (500 MHz, $CDCl_3$)

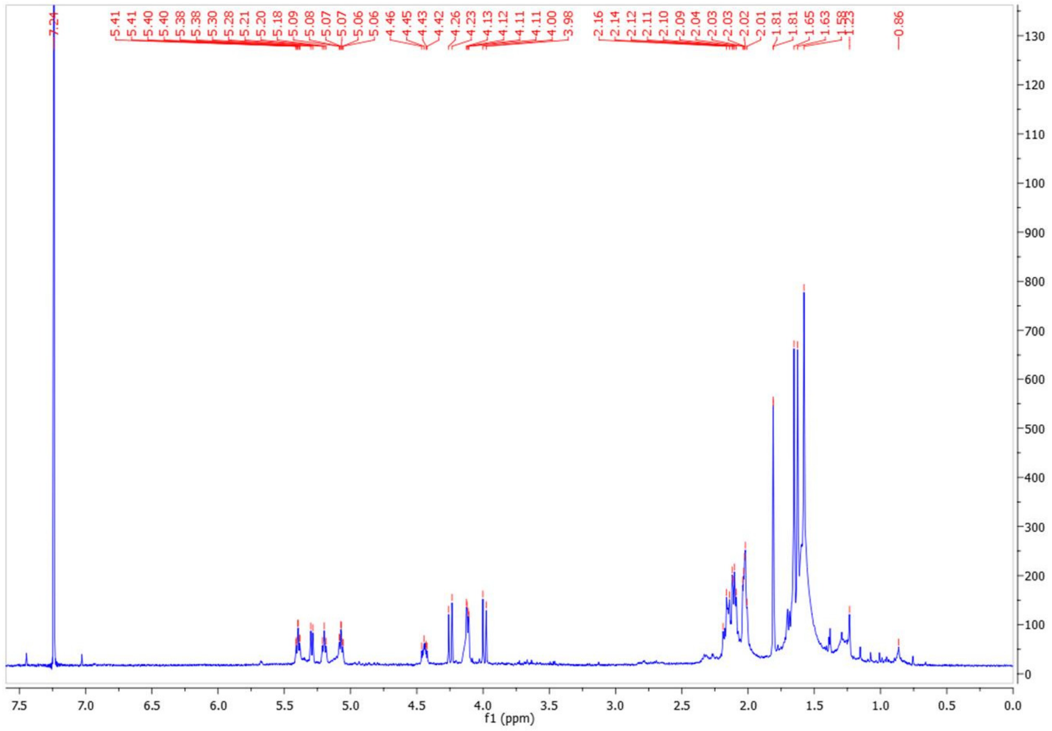

Figure S10. ^{13}C NMR spectrum of compound **2** (125 MHz, $CDCl_3$)

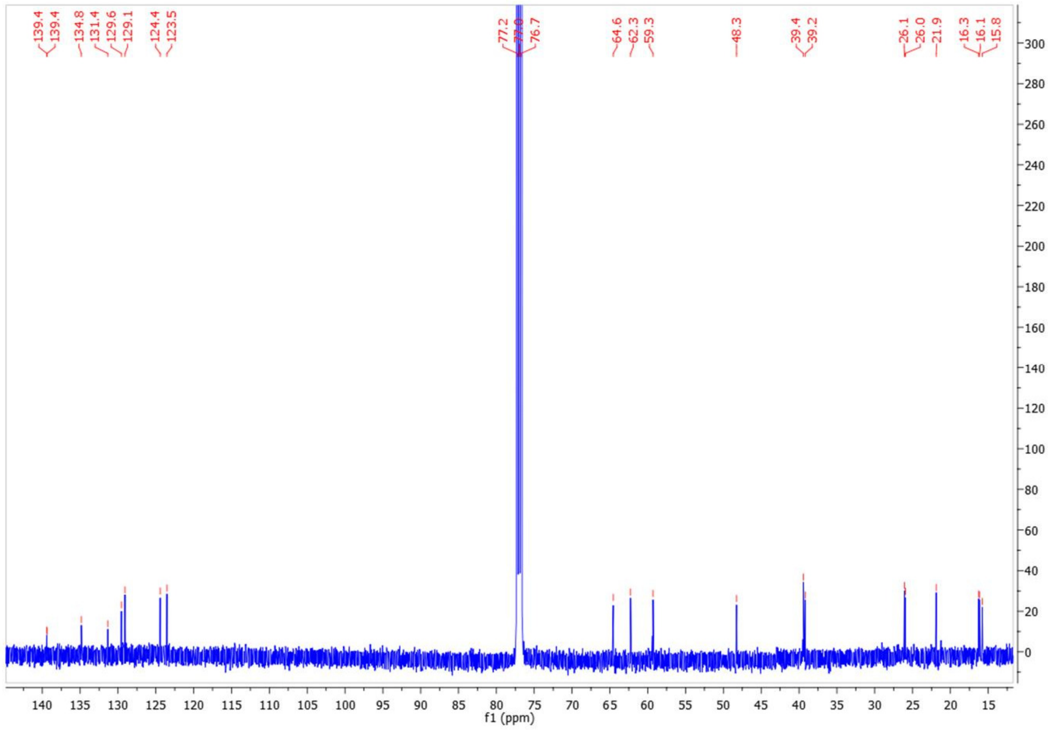

Figure S11. *g*COSY spectrum of compound **2** (500 MHz, $CDCl_3$)

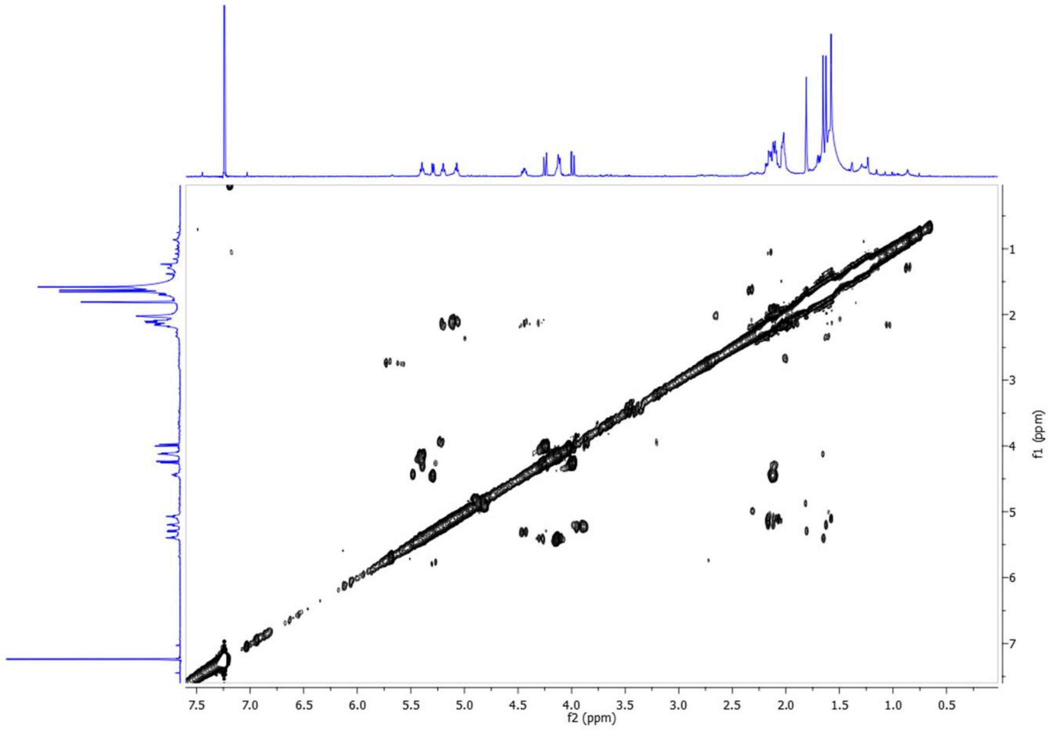

Figure S12. gHSQC spectrum of compound **2** (500/125 MHz, $CDCl_3$)

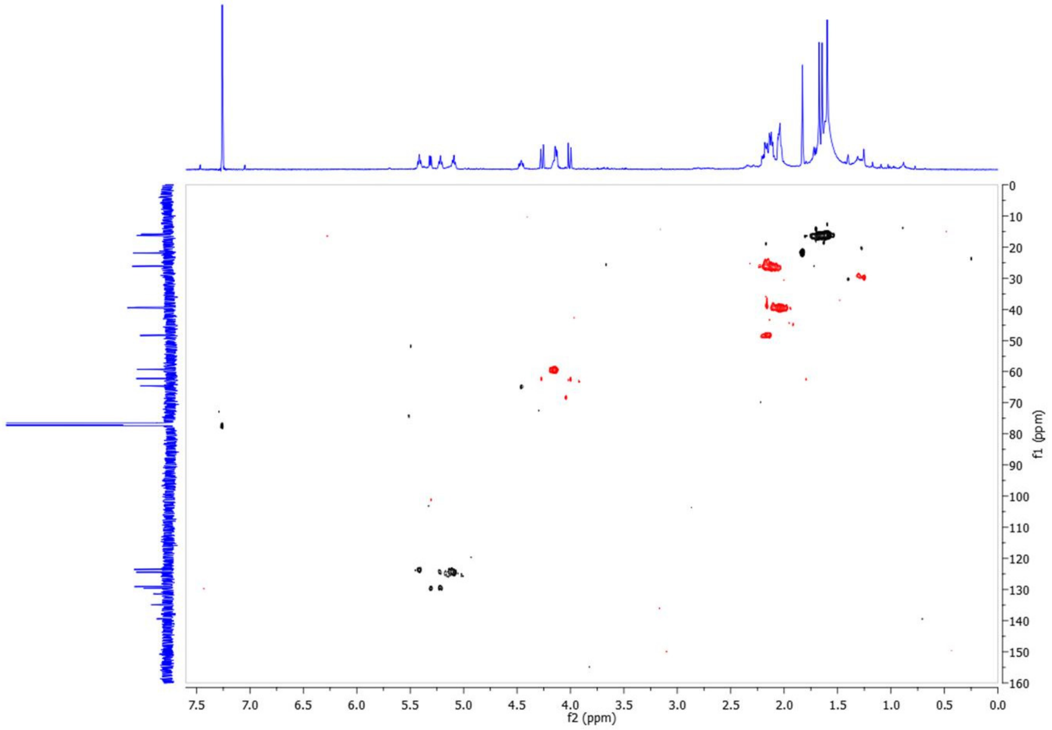

Figure S13. *g*HMBC spectrum of compound **2** (500/125 MHz, $CDCl_3$)

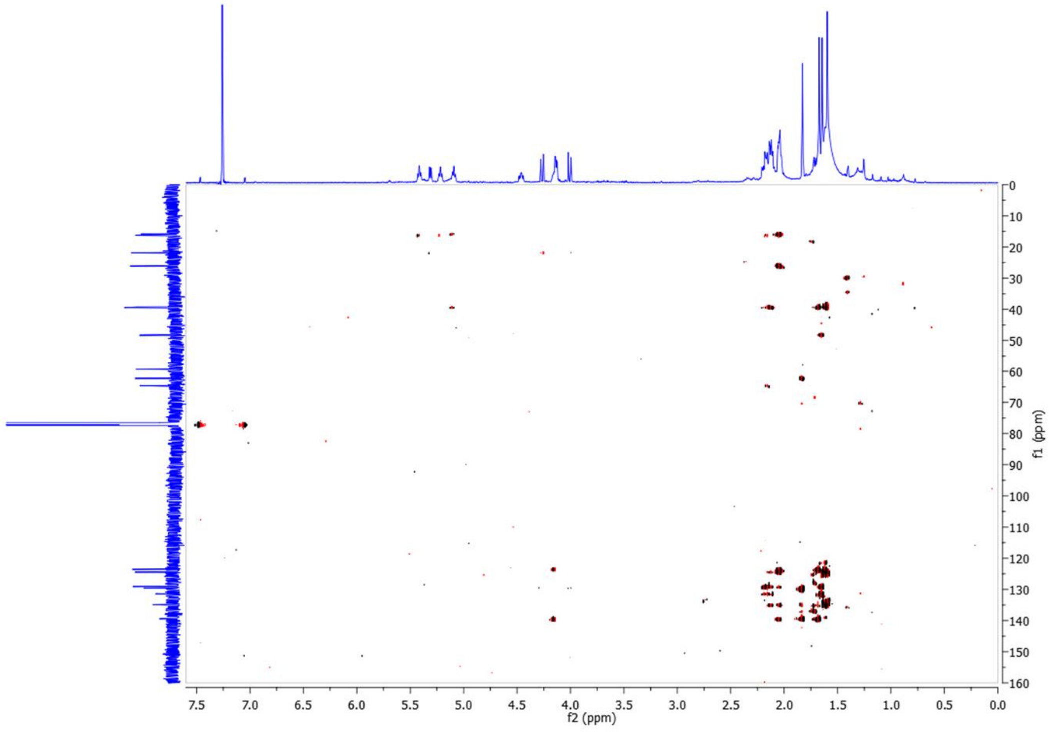

Figure S14. HR-ESIMS report of compound **2**

Qualitative Compound Report

**Data File**	bifu28_15c16-3.d	**Sample Name**	bifu28_15c16-3
**Sample Type**	Sample	**Position**	P1-A9
**Instrument Name**	Instrument 1	**User Name**	
**Acq Method**	VsmyPosFragile_BDV15.m	**Acquired Time**	15-Oct-14 20:10:21
**IRM Calibration Status**	Success	**DA Method**	Default.m
**Comment**			

**Sample Group**	**Info.**
**Fragmentor**	**Nozzle Voltage**

Compound Table

Compound Label	RT	Mass	Abund	Formula	Tgt Mass	Diff (ppm)	MFG Formula	DB Formula
Cpd 1:	6.201	322.2506	961929	C20H34O3	322.2508	-0.76	C20H34O3	C20H34O3

Compound Label	*m/z*	RT	Algorithm	Mass
Cpd 1:	684.474	6.201	Find By Formula	322.2506

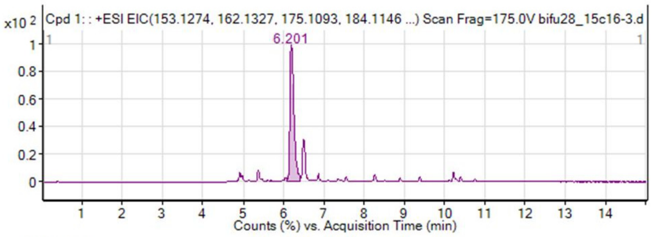

MS Spectrum

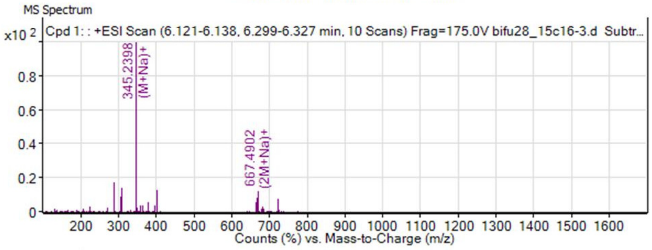

MS Zoomed Spectrum

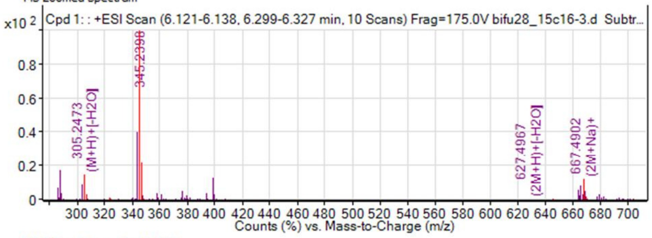

MS Spectrum Peak List

m/z	*Calc m/z*	Diff(ppm)	z	Abund	Formula	Ion
305.2473	305.2475	0.73	1	137608.2	C20H33O2	(M+H)+[-H2O]
323.2574	323.2581	2.11	1	11957.3	C20H35O3	(M+H)+
345.2398	345.24	0.71		961929	C20H34NaO3	(M+Na)+
345.2398			1	961929		
346.2434	346.2434	0.03	1	182610.9	C20H34NaO3	(M+Na)+
627.4967	627.4983	2.55	1	843	C40H67O5	(2M+H)+[-H2O]
645.5082	645.5089	1.08	1	3596.6	C40H69O6	(2M+H)+
649.4733	649.4802	10.73	1	151.8	C40H66NaO5	(2M+Na)+[-H2O]
667.4902	667.4908	0.93	1	118466.1	C40H68NaO6	(2M+Na)+
683.4717	683.4647	-10.18	1	3503.9	C40H68KO6	(2M+K)+

--- End Of Report ---

Figure S15. FT-IR spectrum of compound **2**

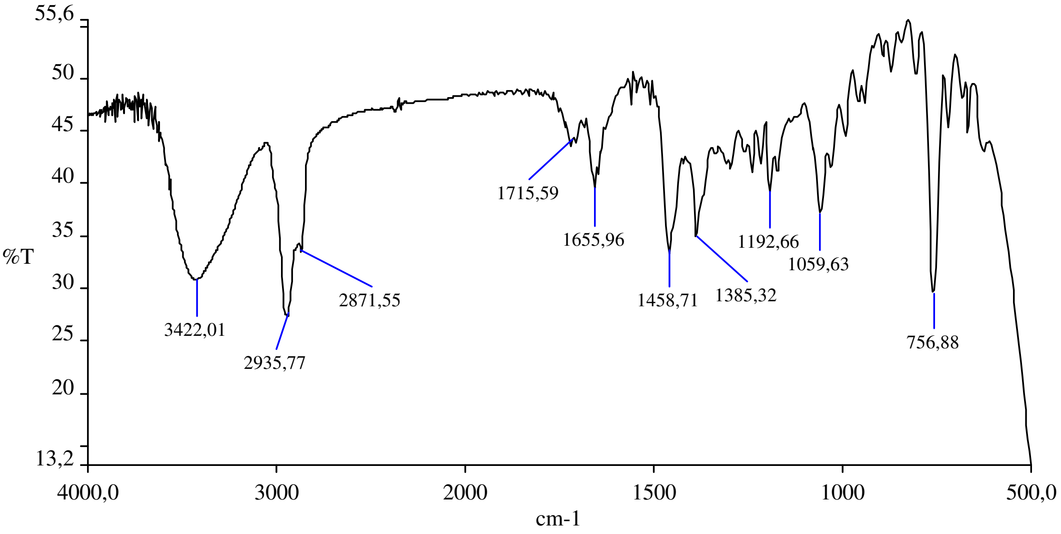

Figure S16. ^{1}H NMR spectrum of compound **3** (600 MHz, C_6D_6)

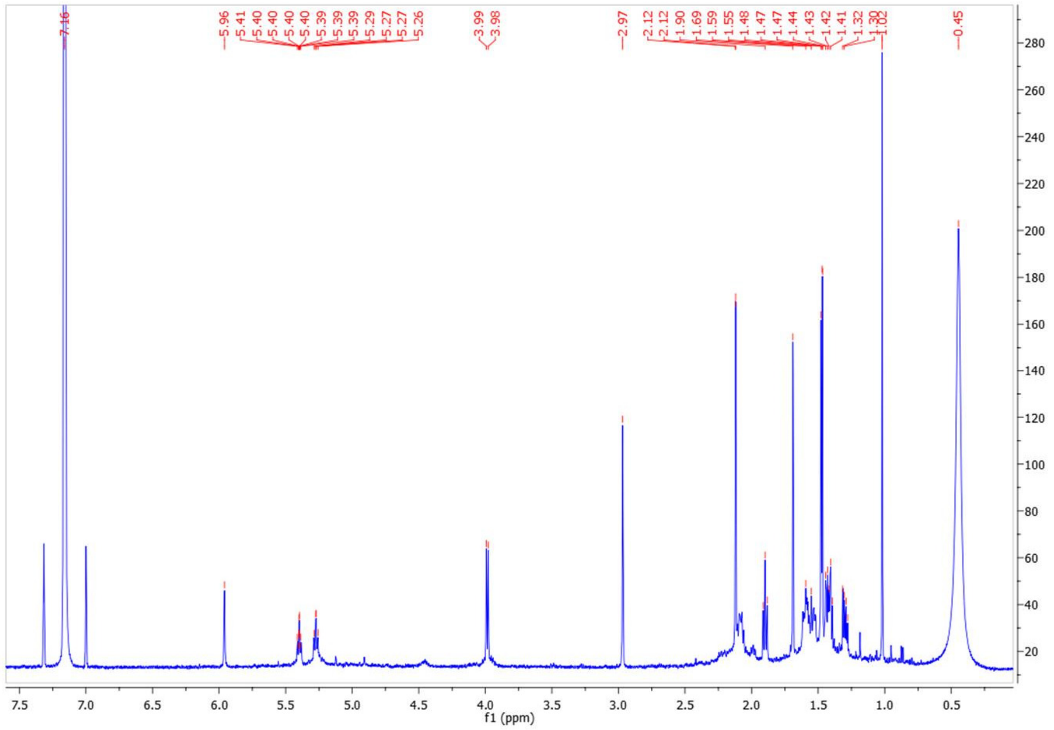

Figure S17. ^{13}C NMR spectrum of compound **3** (150 MHz, C_6D_6)

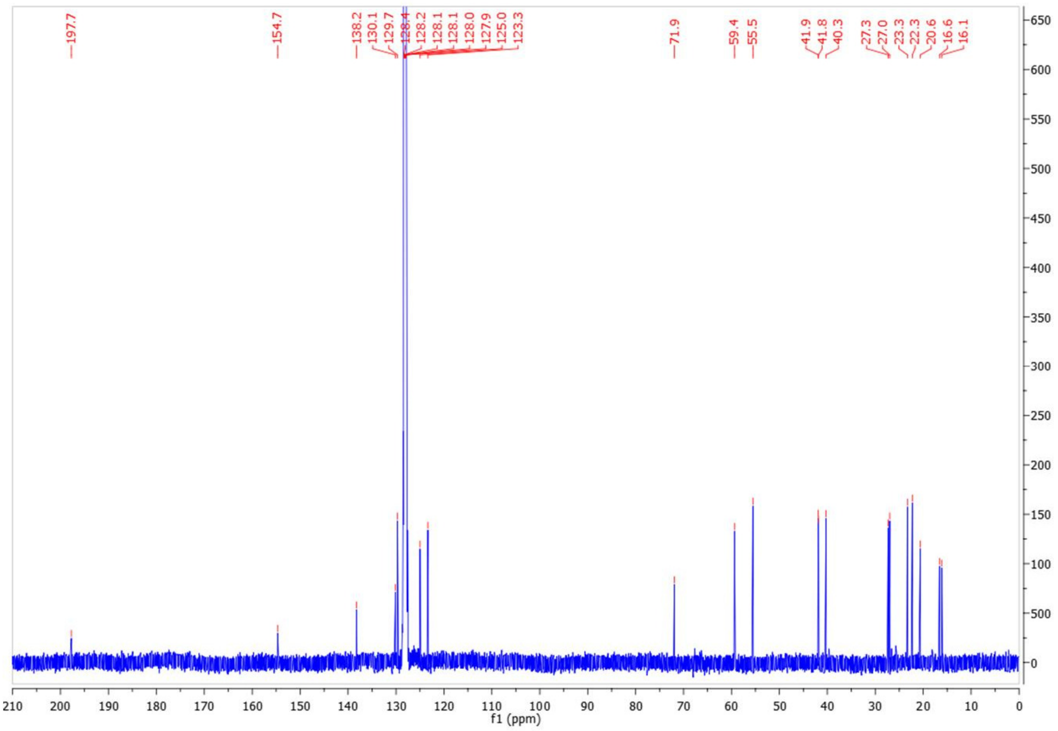

Figure S18. *g*COSY spectrum of compound **3** (600 MHz, C_6D_6)

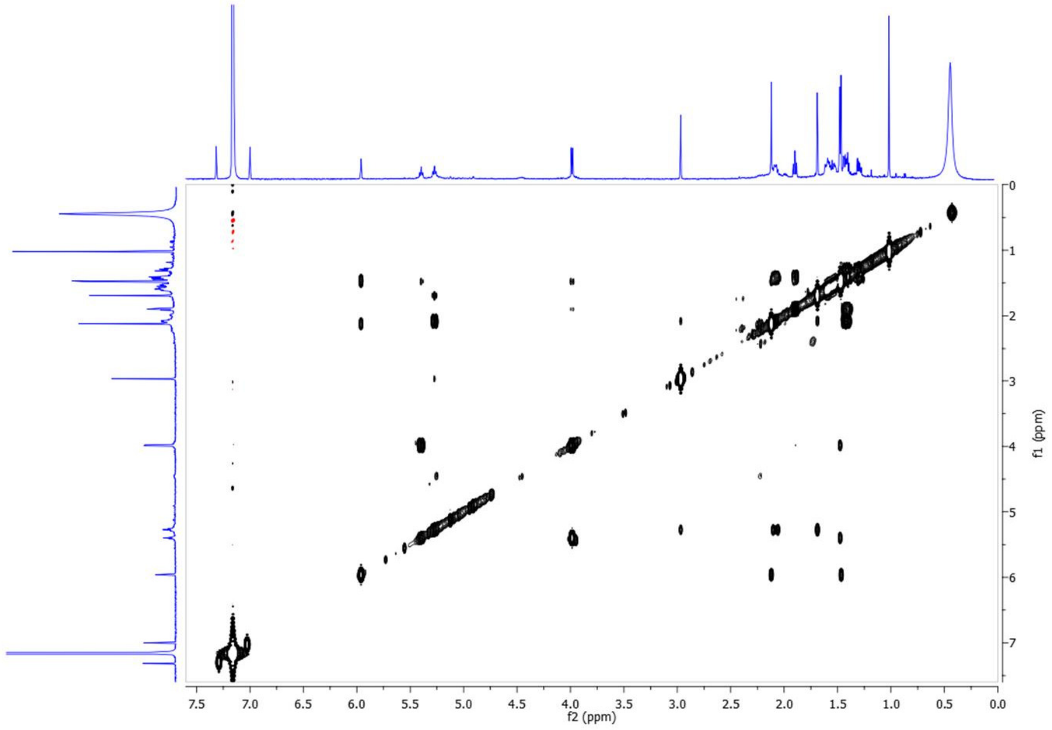

Figure S19. gHSQC spectrum of compound **3** (600/150 MHz, C_6D_6)

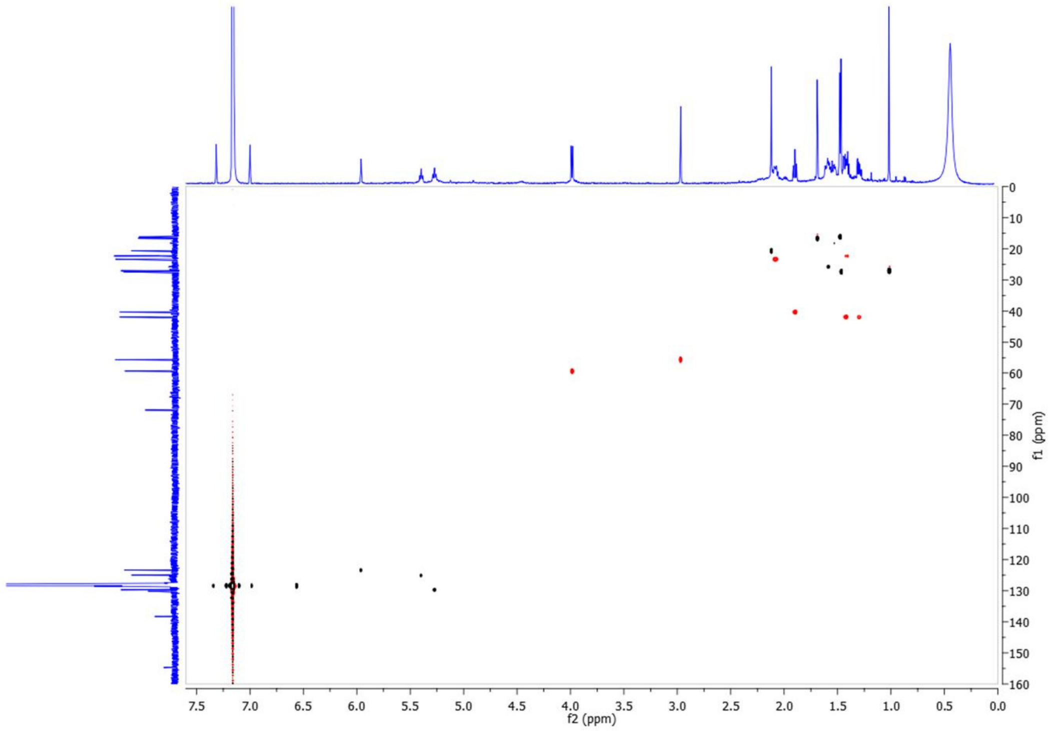

Figure S20. *g*HMBC spectrum of compound **3** (600/150 MHz, C_6D_6)

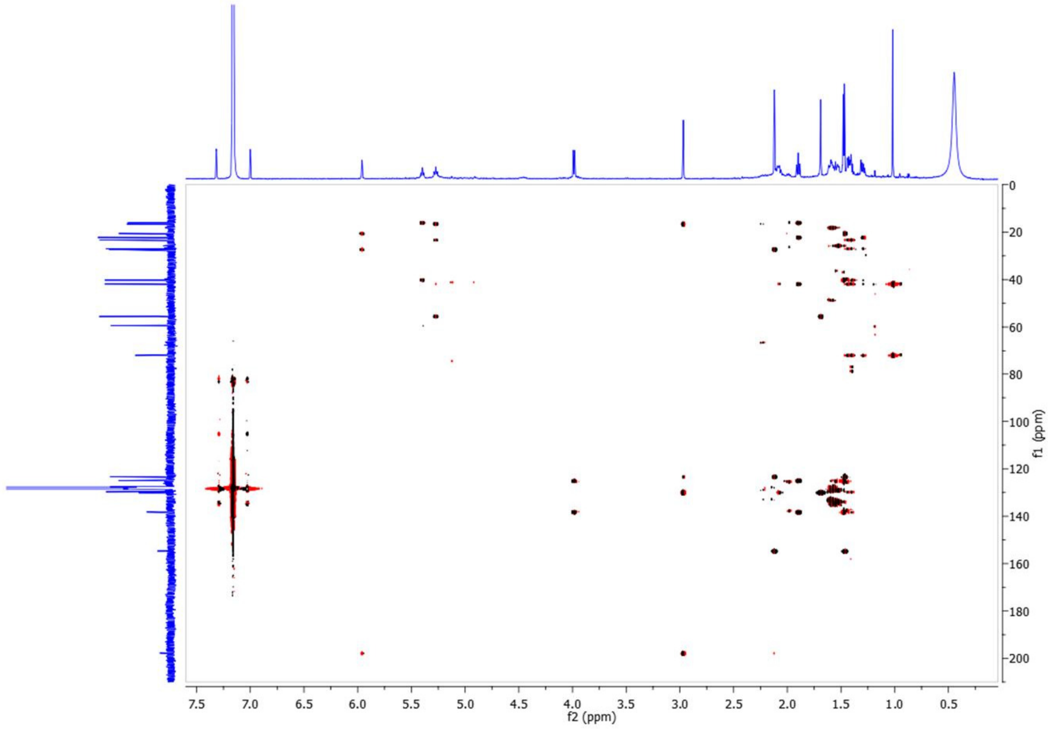

Figure S21. NOESY spectrum of compound **3** (500 MHz, C_6D_6)

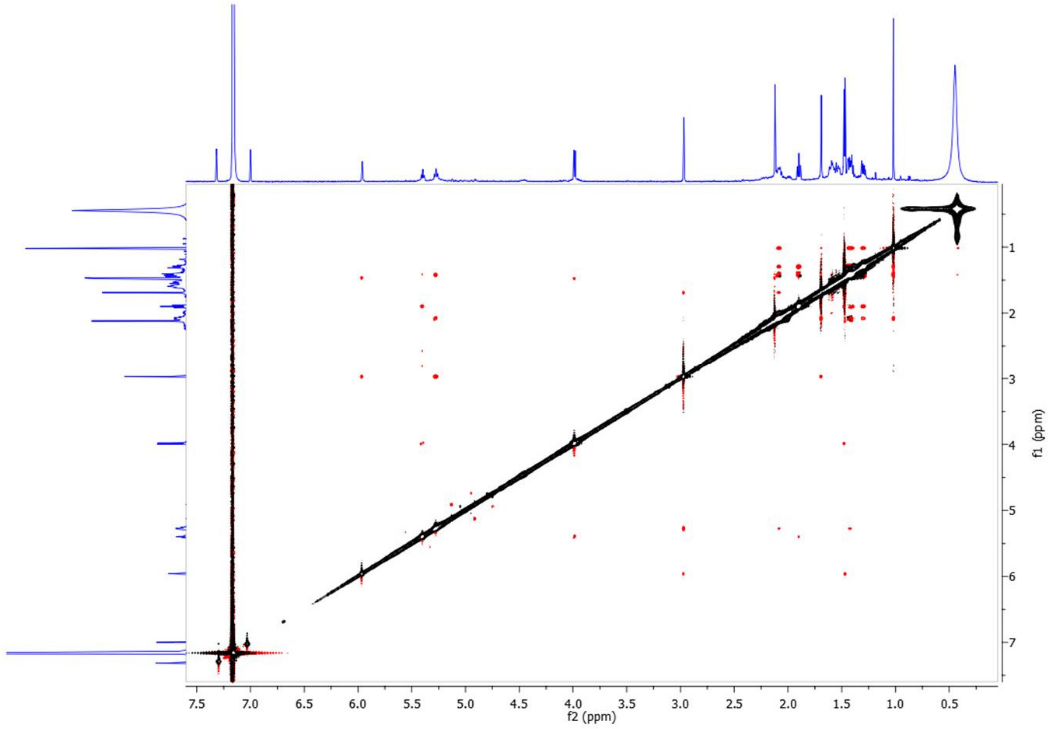

Figure S22. HR-ESIMS report of compound **3**

Qualitative Compound Report

**Data File**	bifu25_15c12-5.d	**Sample Name**	bifu25_15c12-5
**Sample Type**	Sample	**Position**	P1-A2
**Instrument Name**	Instrument 1	**User Name**	
**Acq Method**	VsmyPosFragile_BDV15.m	**Acquired Time**	14-Oct-14 00:57:32
**IRM Calibration Status**	Success	**DA Method**	Default.m
**Comment**			

**Sample Group**	**Info.**
**Fragmentor**	**Nozzle Voltage**

Compound Table

Compound Label	RT	Mass	Abund	Formula	Tgt Mass	Diff (ppm)	MFG Formula	DB Formula
Cpd 1:	6.041	322.251	1288015	C20H34O3	322.2508	0.52	C20H34O3	C20H34O3

Compound Label	*m/z*	RT	Algorithm	Mass
Cpd 1:	648.518	6.041	Find By Formula	322.251

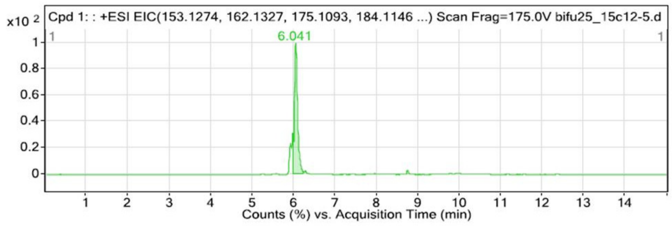

MS Spectrum

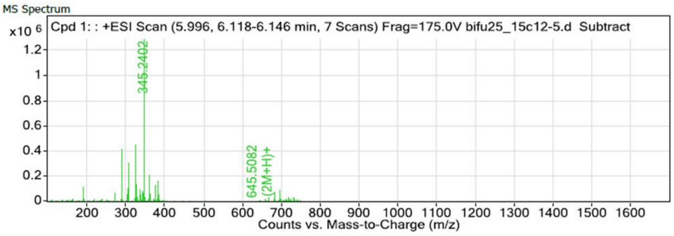

MS Zoomed Spectrum

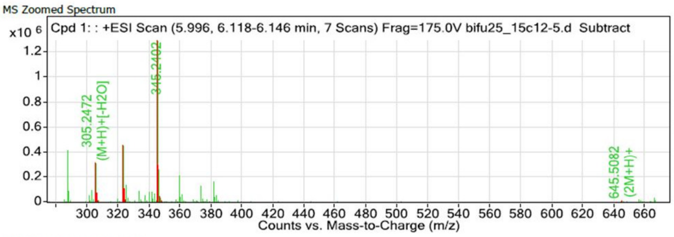

MS Spectrum Peak List

m/z	*Calc m/z*	Diff(ppm)	z	Abund	Formula	Ion
305.2472	305.2475	-1.04	1	306579.3	C20H33O2	(M+H)+[-H2O]
306.2507	306.2509	-0.83	1	62820.2	C20H33O2	(M+H)+[-H2O]
307.257	307.2539	10.03	1	10958.1	C20H33O2	(M+H)+[-H2O]
323.257	323.2581	-3.3	1	194468.1	C20H35O3	(M+H)+
324.2602	324.2615	-4.02	1	36451.9	C20H35O3	(M+H)+
345.2402				1288015.1		
345.2402	345.24	0.53	1	1288015.1	C20H34NaO3	(M+Na)+
346.2432	346.2434	-0.65	1	259686.5	C20H34NaO3	(M+Na)+
347.2469	347.2463	1.65	1	34581.2	C20H34NaO3	(M+Na)+
645.5082	645.5089	-1.06	1	5336.3	C40H69O6	(2M+H)+

--- End Of Report ---

Figure S23. FT-IR spectrum of compound **3**

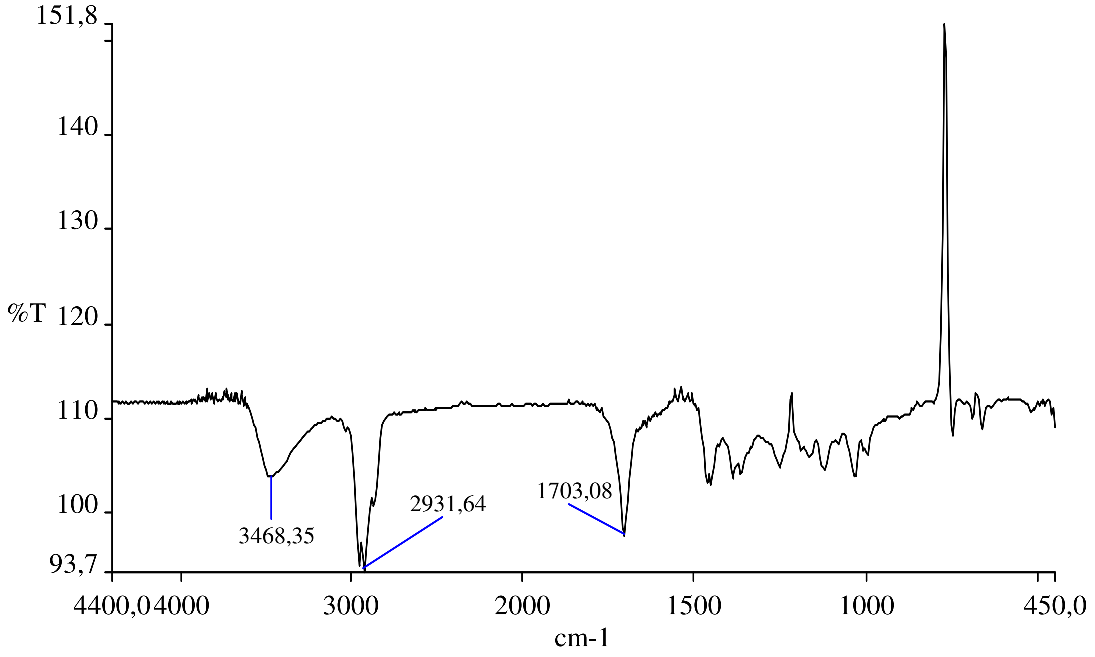

Figure S24. ¹H NMR spectrum of compound **4** (500 MHz, $CDCl_3$)

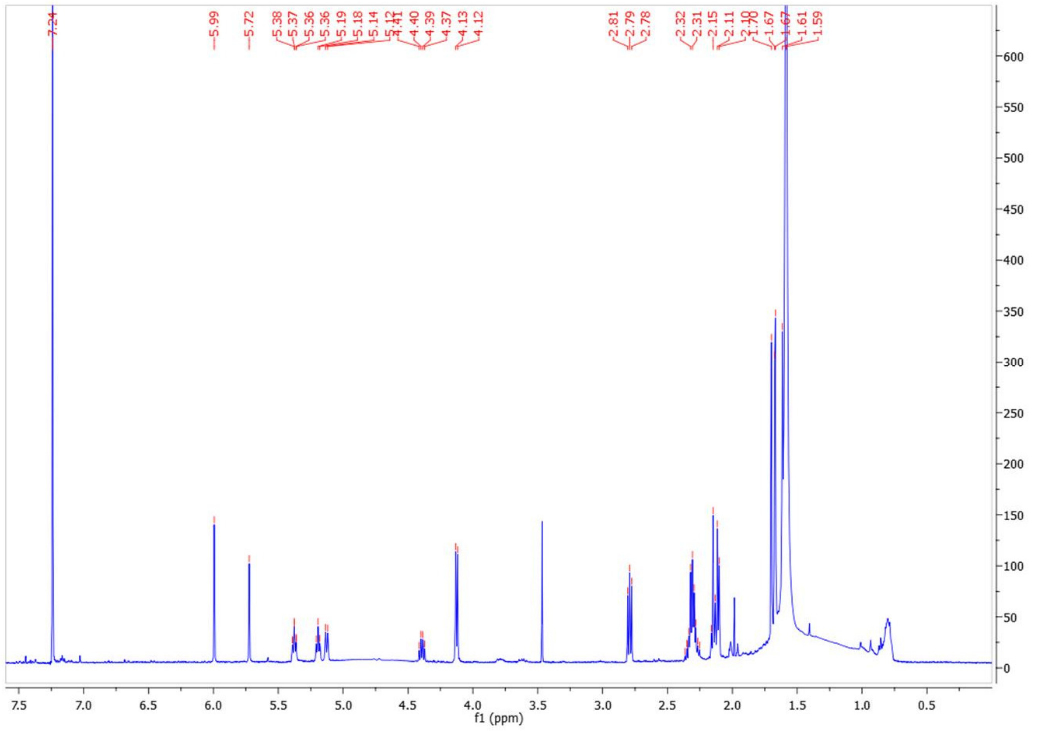

Figure S25. *g*HSQC spectrum of compound **4** (500/125 MHz, $CDCl_3$)

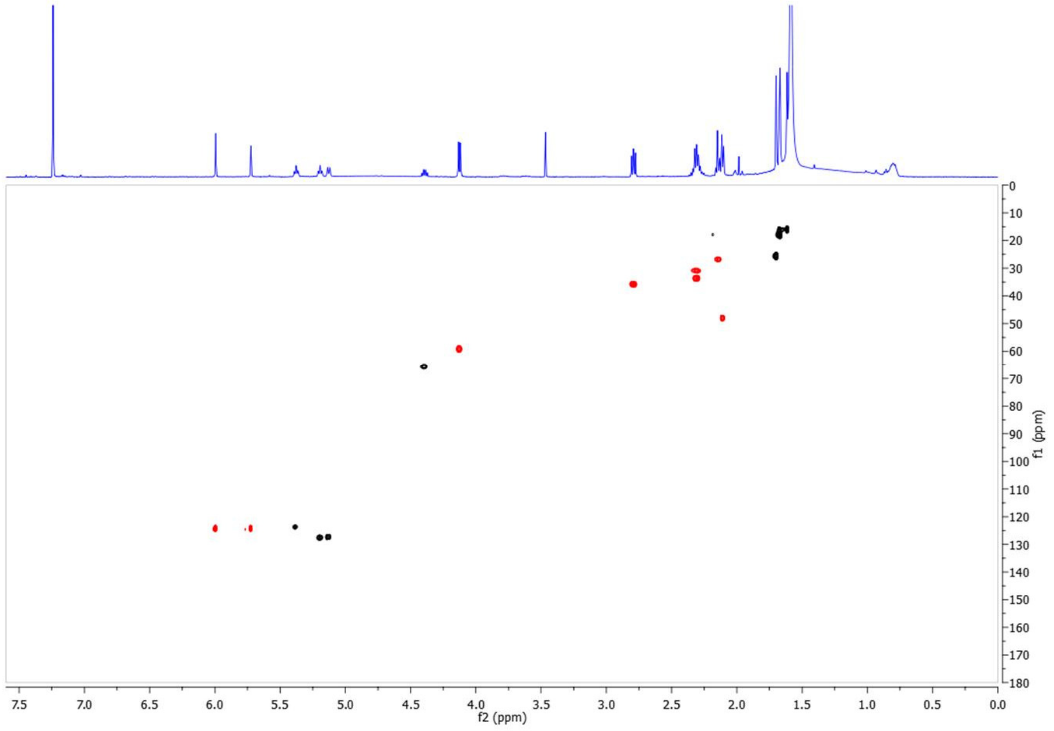

Figure S26. *g*COSY spectrum of compound **4** (500 MHz, $CDCl_3$)

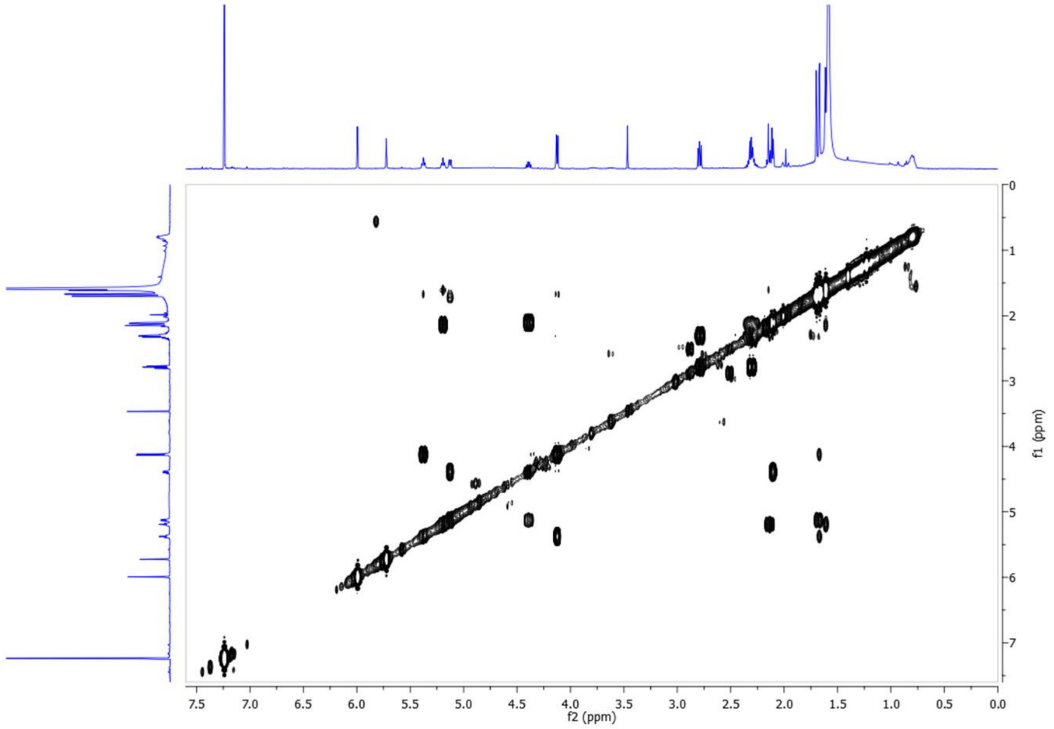

Figure S27. *g*HMBC spectrum of compound **4** (500/125 MHz, $CDCl_3$)

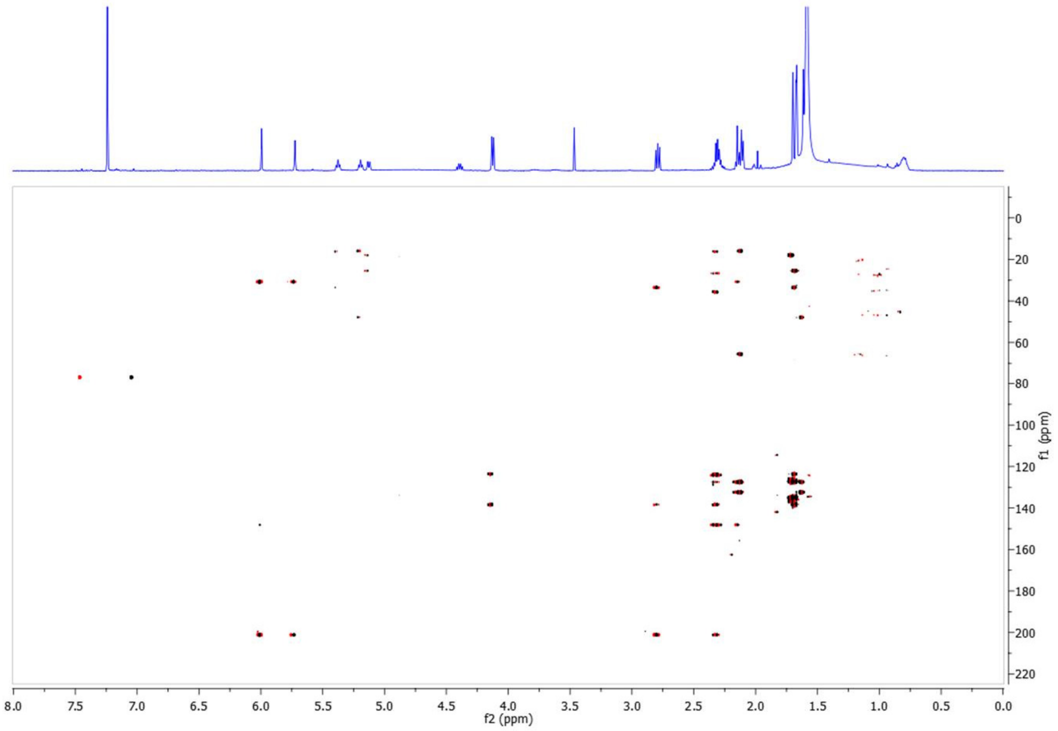

Figure S28. HR-ESIMS report of compound **4**

Qualitative Compound Report

**Data File**	bifu18_15C10-5.d	**Sample Name**	15C10-5_bifu18
**Sample Type**	Sample	**Position**	P1-A9
**Instrument Name**	Instrument 1	**User Name**	
**Acq Method**	VsmyPosFragile_BDV15.m	**Acquired Time**	30-Jul-14 20:28:18
**IRM Calibration Status**	Success	**DA Method**	Default.m
**Comment**			

**Sample Group**	**Info.**
**Fragmentor**	**Nozzle Voltage**

Compound Table

Compound Label	RT	Mass	Abund	Formula	Tgt Mass	Diff (ppm)	MFG Formula	DB Formula
Cpd 1:	7.335	320.2354	223046	C20H32O3	320.2351	0.74	C20H32O3	C20H32O3

Compound Label	*m/z*	RT	Algorithm	Mass
Cpd 1:	986.6763	7.335	Find By Formula	320.2354

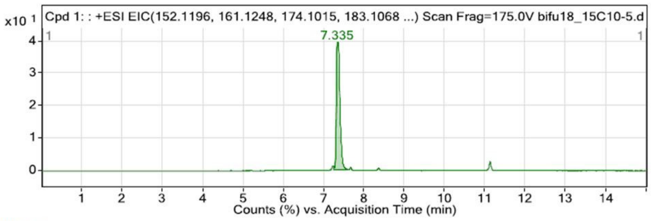

MS Spectrum

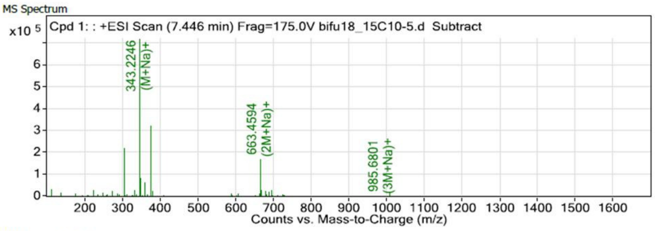

MS Zoomed Spectrum

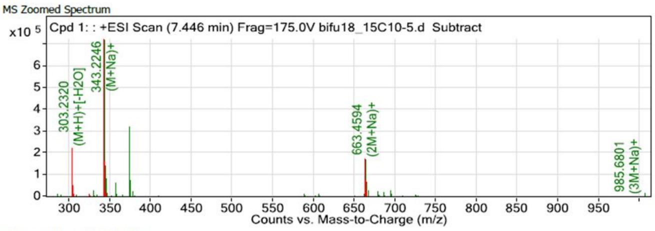

MS Spectrum Peak List

m/z	*Calc m/z*	Diff(ppm)	z	Abund	Formula	Ion
303.232	303.2319	0.43	1	223045.8	C20H31O2	(M+H)+[-H2O]
321.2403	321.2424	-6.57	1	569.8	C20H33O3	(M+H)+
325.2133	325.2138	-1.57	1	7700	C20H30NaO2	(M+Na)+[-H2O]
343.2246				719350.6		
343.2246	343.2244	0.66	1	719350.6	C20H32NaO3	(M+Na)+
344.2284	344.2278	1.79	1	143577.2	C20H32NaO3	(M+Na)+
641.4728	641.4776	-7.43	1	374.8	C40H65O6	(2M+H)+
645.4471	645.4489	-2.85	1	486.4	C40H62NaO5	(2M+Na)+[-H2O]
663.4594	663.4595	-0.2	1	170280.5	C40H64NaO6	(2M+Na)+
983.6875	983.6947	-7.27	1	561.4	C60H96NaO9	(3M+Na)+

--- End Of Report ---

Figure S29. FT-IR spectrum of compound **4**

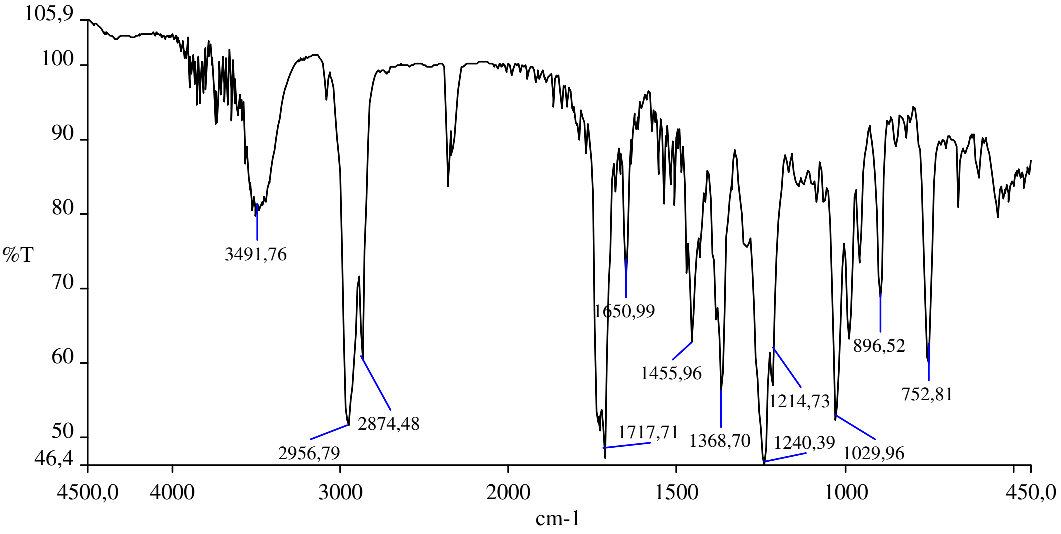

Figure S30. ^{1}H NMR spectrum of compound **5** (500 MHz, $CDCl_3$)

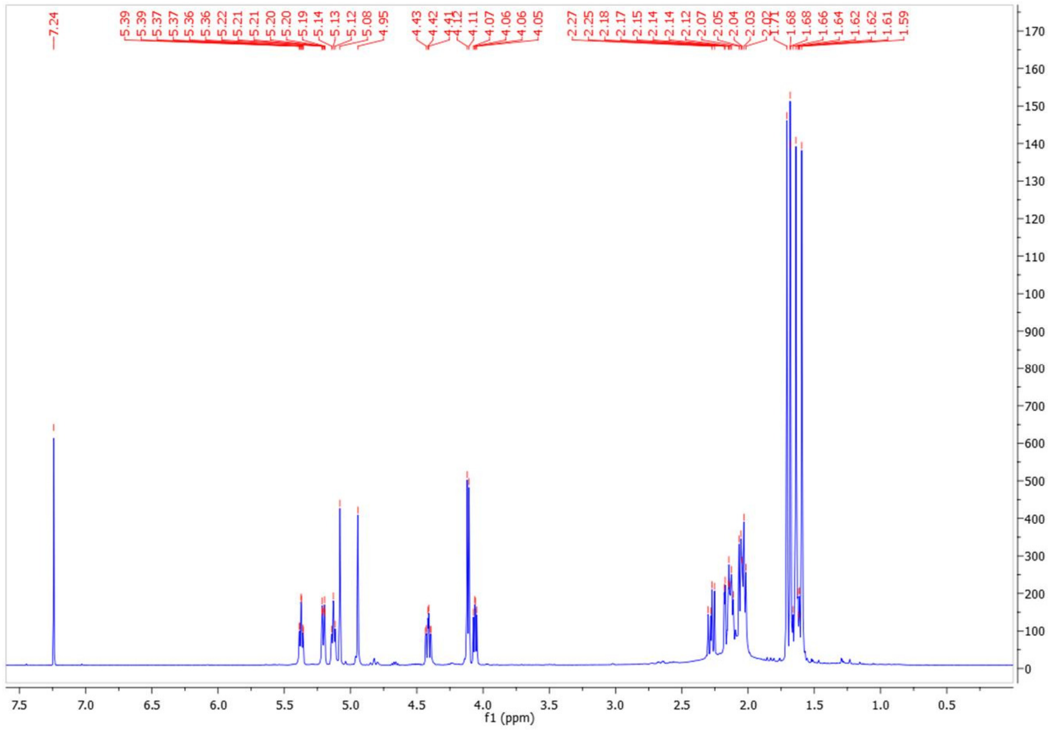

Figure S31. ^{13}C NMR spectrum of compound **5** (125 MHz, $CDCl_3$)

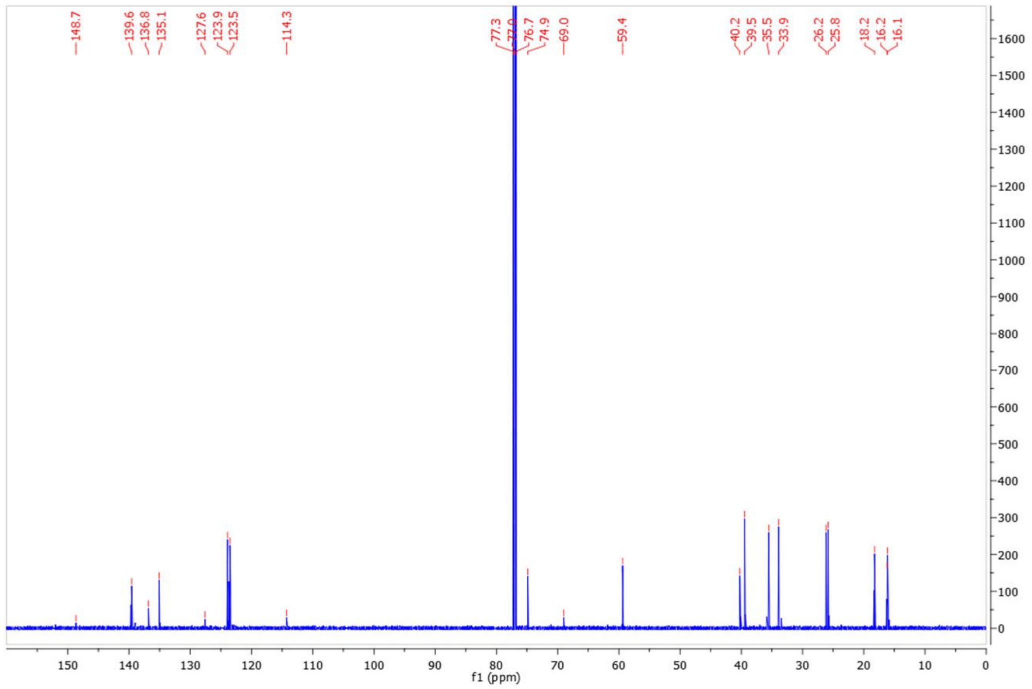

Figure S32. *g*HSQC spectrum of compound **5** (500/125 MHz, $CDCl_3$)

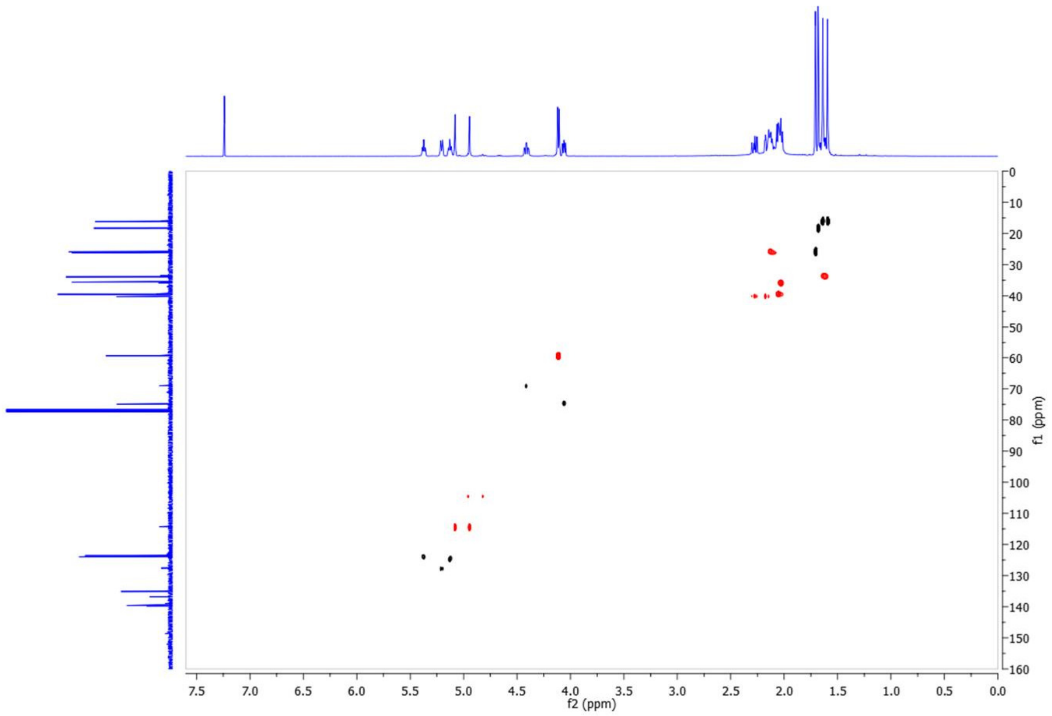

Figure S33. *g*HMBC spectrum of compound **5** (500/125 MHz, $CDCl_3$)

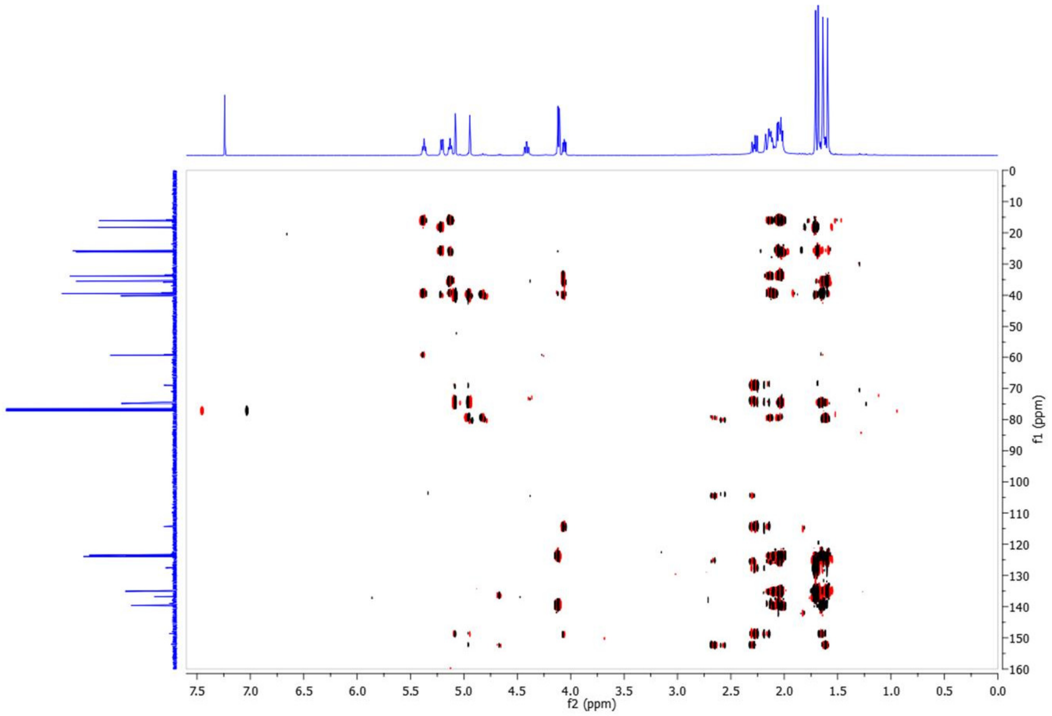

Figure S34. HR-ESIMS report of compound **5**

Qualitative Compound Report

**Data File**	bifu26_15c12-6.d	**Sample Name**	bifu26_15c12-6
**Sample Type**	Sample	**Position**	P1-A8
**Instrument Name**	Instrument 1	**User Name**	
**Acq Method**	VsmyPosFragile_BDV15.m	**Acquired Time**	16-Oct-14 08:11:15
**IRM Calibration Status**	Success	**DA Method**	Default.m
**Comment**			

**Sample Group**	**Info.**
**Fragmentor**	**Nozzle Voltage**

Compound Table

Compound Label	RT	Mass	Abund	Formula	Tgt Mass	Diff (ppm)	MFG Formula	DB Formula
Cpd 1:	6.423	322.2512	2E+06	C20H34O3	322.2508	1.25	C20H34O3	C20H34O3

Compound Label	*m/z*	RT	Algorithm	Mass
Cpd 1:	991.7195	6.423	Find By Formula	322.2512

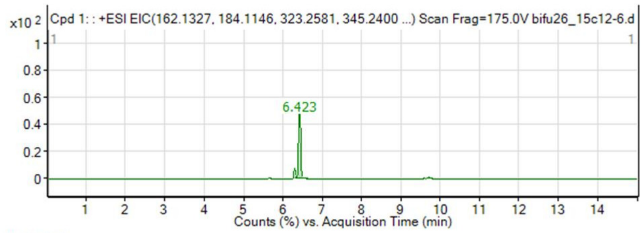

MS Spectrum

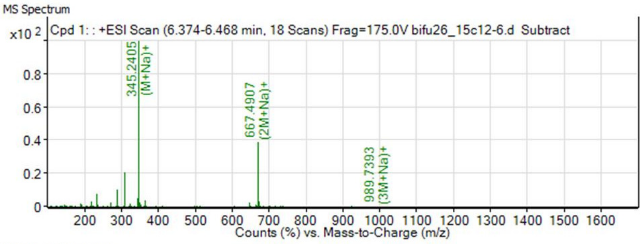

MS Zoomed Spectrum

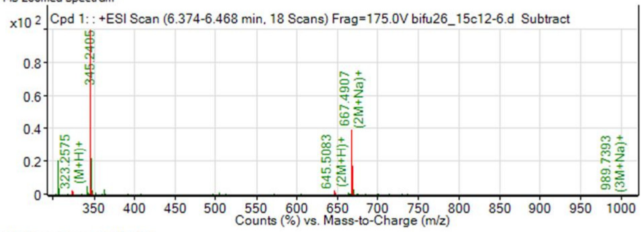

MS Spectrum Peak List

m/z	*Calc m/z*	Diff(ppm)	z	Abund	Formula	Ion
323.2575	323.2581	1.87	1	38343.7	C20H35O3	(M+H)+
345.2405	345.24	-1.28		1924244.3	C20H34NaO3	(M+Na)+
345.2405			1	1924244.3		
346.2432	346.2434	0.59	1	423490.7	C20H34NaO3	(M+Na)+
347.2463	347.2463	0.15	1	52222.5	C20H34NaO3	(M+Na)+
645.5083	645.5089	0.82	1	45333.3	C40H69O6	(2M+H)+
667.4907	667.4908	0.19	1	753009.1	C40H68NaO6	(2M+Na)+
668.494	668.4942	0.32	1	311111.5	C40H68NaO6	(2M+Na)+
669.4969	669.4973	0.63	1	72468.4	C40H68NaO6	(2M+Na)+
989.7393	989.7416	2.3	1	233.1	C60H102NaO9	(3M+Na)+

--- End Of Report ---

Figure S35. FT-IR spectrum of compound **5**

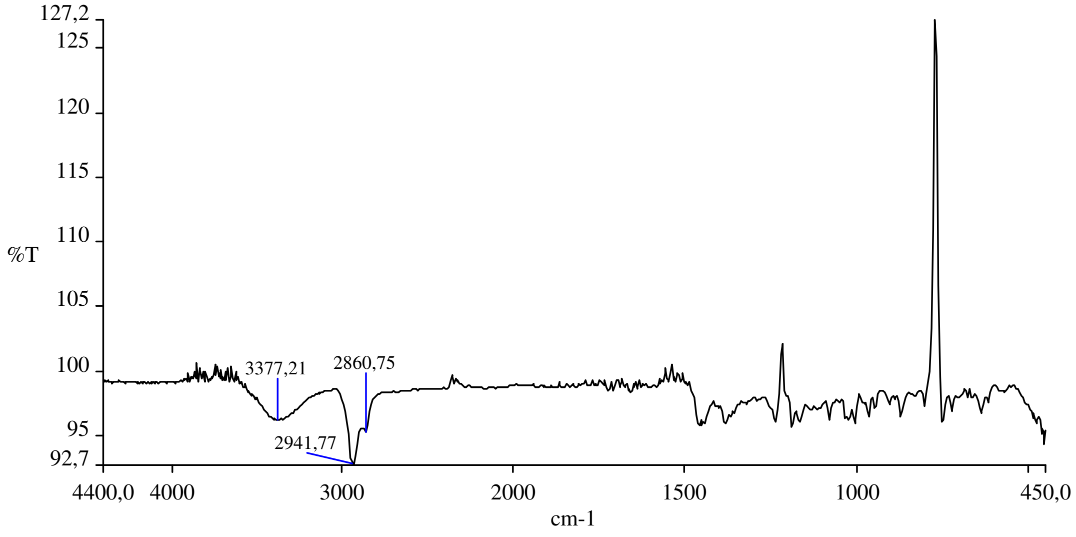

Figure S36. ^{1}H NMR spectrum of compound **6** (600 MHz, $CDCl_3$)

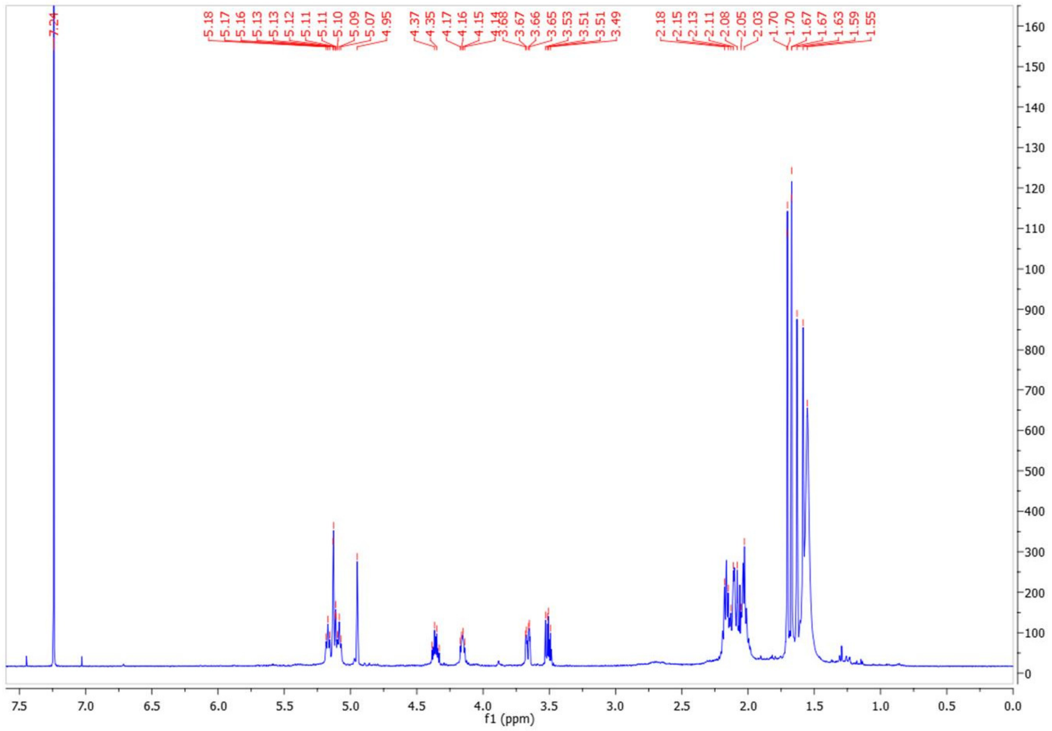

Figure S37. ^{13}C NMR spectrum of compound **6** (150 MHz, $CDCl_3$)

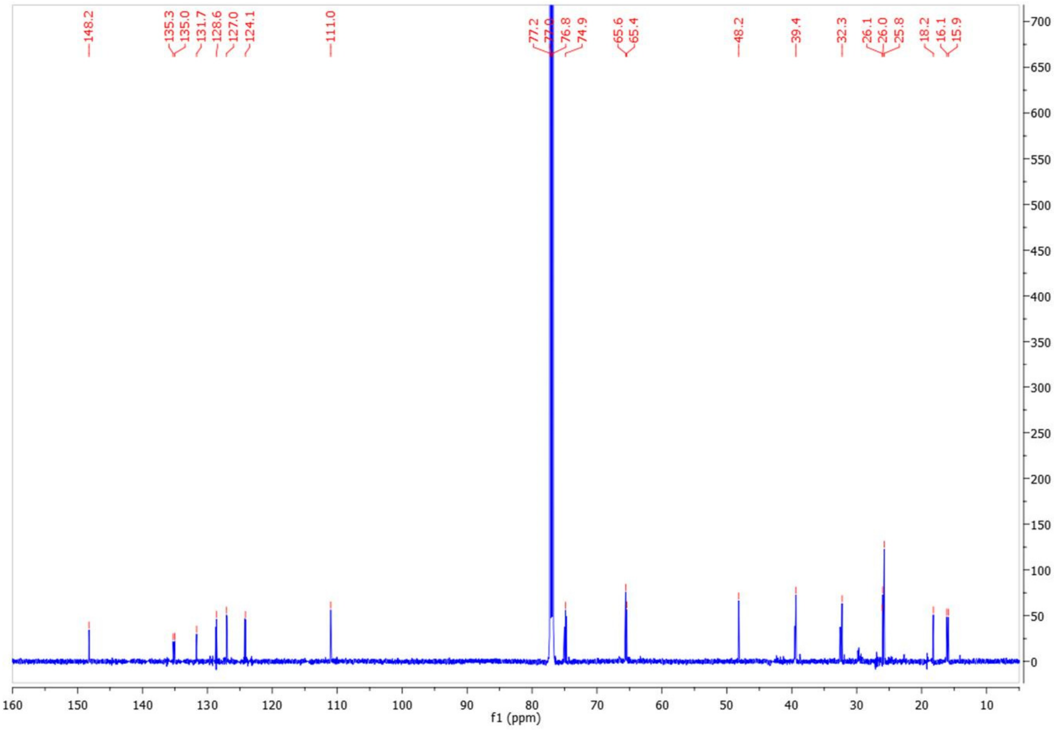

Figure S38. gCOSY spectrum of compound **6** (600 MHz, $CDCl_3$)

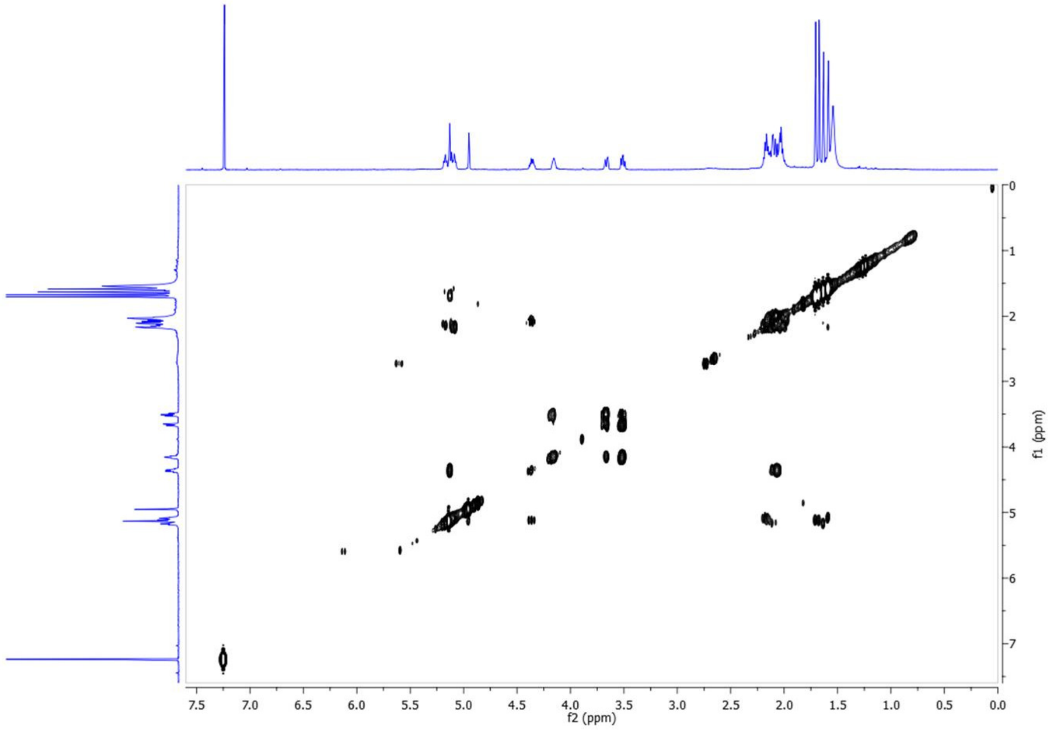

Figure S39. *g*HMBC spectrum of compound **6** (600/150 MHz, $CDCl_3$)

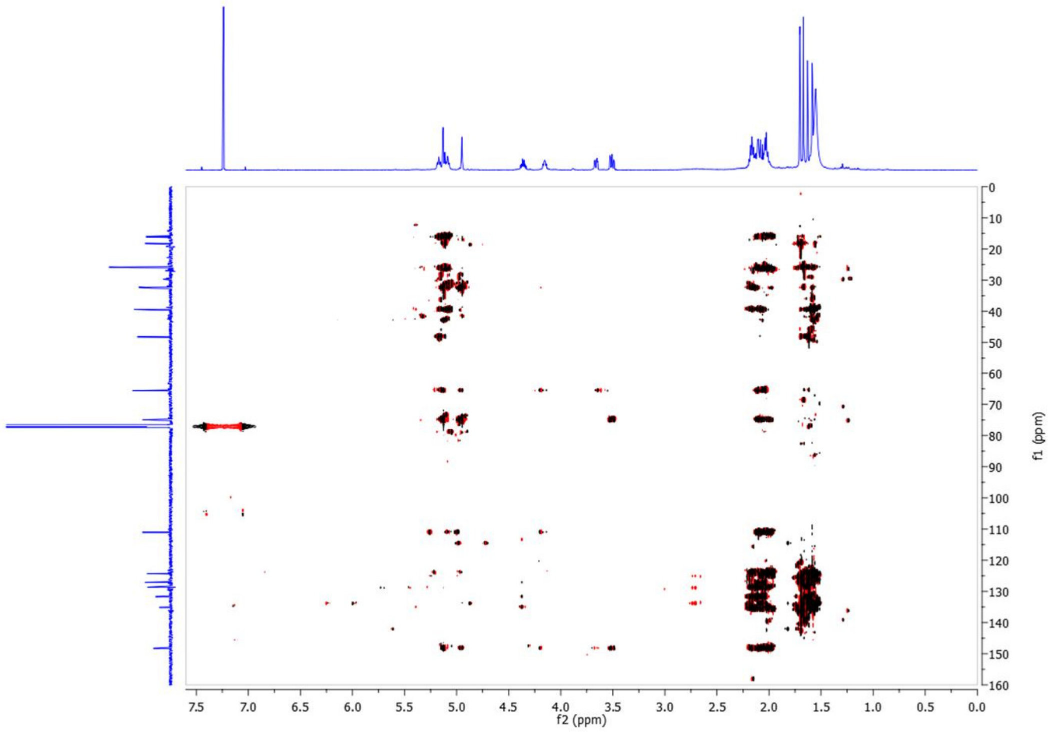

Figure S40. HR-ESIMS report of compound **6**

Qualitative Compound Report

**Data File**	bifu27_15c12-7.d	**Sample Name**	bifu27_15c12-7
**Sample Type**	Sample	**Position**	P1-A9
**Instrument Name**	Instrument 1	**User Name**	
**Acq Method**	VsmyPosFragile_BDV15.m	**Acquired Time**	16-Oct-14 08:47:28
**IRM Calibration Status**	Success	**DA Method**	Default.m
**Comment**			

**Sample Group**	**Info.**
**Fragmentor**	**Nozzle Voltage**

Compound Table

Compound Label	RT	Mass	Abund	Formula	Tgt Mass	Diff (ppm)	MFG Formula	DB Formula
Cpd 1:	6.529	322.2504	970474	C20H34O3	322.2508	-1.25	C20H34O3	C20H34O3

Compound Label	*m/z*	RT	Algorithm	Mass
Cpd 1:	671.503	6.529	Find By Formula	322.2504

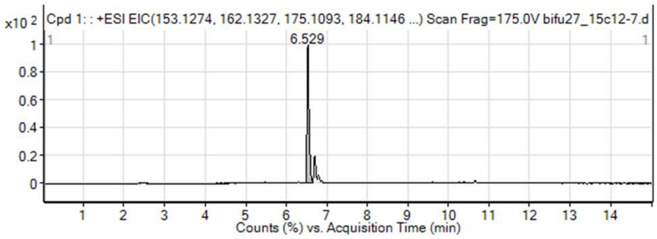

MS Spectrum

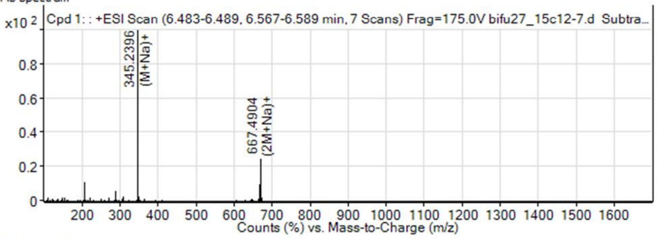

MS Zoomed Spectrum

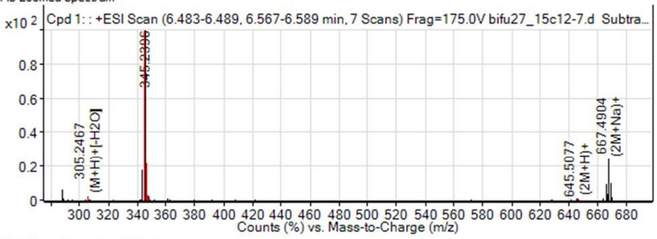

MS Spectrum Peak List

m/z	*Calc m/z*	Diff(ppm)	z	Abund	Formula	Ion
305.2467	305.2475	-2.59	1	25802.9	C20H33O2	(M+H)+[-H2O]
323.257	323.2581	-3.24	1	2788.4	C20H35O3	(M+H)+
345.2396				970473.5		
345.2396	345.24	-1.17	1	970473.5	C20H34NaO3	(M+Na)+
346.2432	346.2434	-0.63	1	189039.9	C20H34NaO3	(M+Na)+
627.4974	627.4983	-1.45	1	2920.2	C40H67O5	(2M+H)+[-H2O]
645.5077	645.5089	-1.83	1	10239.8	C40H69O6	(2M+H)+
649.4791	649.4802	-1.74	1	360.8	C40H66NaO5	(2M+Na)+[-H2O]
667.4904	667.4908	-0.66	1	240588.8	C40H68NaO6	(2M+Na)+
668.4938	668.4942	-0.65	1	99788.4	C40H68NaO6	(2M+Na)+

--- End Of Report ---

Figure S41. FT-IR spectrum of compound **6**

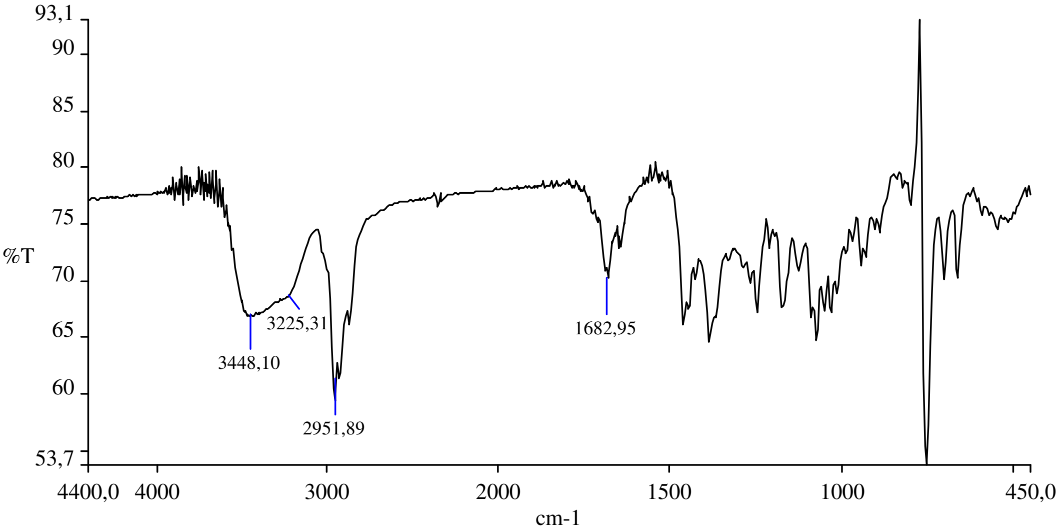

Figure S42. (**a**) Comparison of the IR/VCD spectra of **1** with those of eleganediol (**9**) and calculations for a fragment. (**b**) Fragment used in the calculations that has been shown before (*Chem. Commun.*, **2015**, *51*, 16217) to be sufficient to describe the observed VCD signatures of eleganediol (**9**).

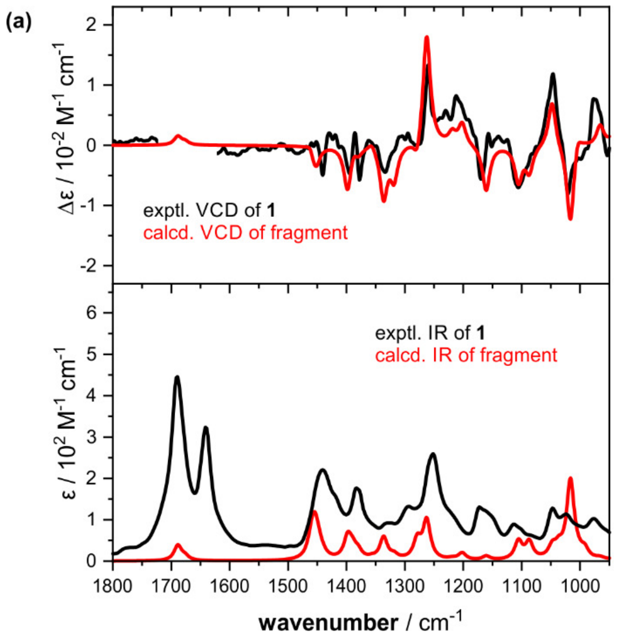

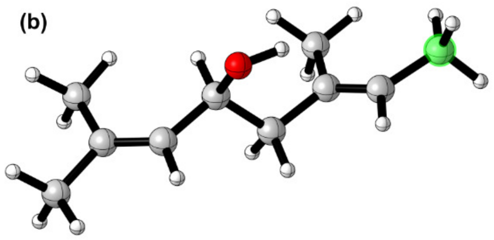

Supplement: Supplementary file 1 [file marinedrugs-18-00581-s001.pdf]
